# Three-dimensional chromatin interactions remain stable upon CAG/CTG repeat expansion

**DOI:** 10.1126/sciadv.aaz4012

**Published:** 2020-07-03

**Authors:** Gustavo A. Ruiz Buendía, Marion Leleu, Flavia Marzetta, Ludovica Vanzan, Jennifer Y. Tan, Victor Ythier, Emma L. Randall, Ana C. Marques, Tuncay Baubec, Rabih Murr, Ioannis Xenarios, Vincent Dion

**Affiliations:** 1 Center for Integrative Genomics, Faculty of Biology and Medicine, University of Lausanne, 1015 Lausanne, Switzerland.; 2 School of Life Sciences, Ecole Polytechnique Fédérale de Lausanne, 1015 Lausanne, Switzerland.; 3 Vital-IT Group, Swiss Institute of Bioinformatics, 1015 Lausanne, Switzerland.; 4 Department of Genetic Medicine and Development, University of Geneva Medical School, 1211 Geneva, Switzerland.; 5 Department of Computational Biology, Faculty of Biology and Medicine, University of Lausanne, 1015 Lausanne, Switzerland.; 6 UK Dementia Research Institute at Cardiff University at Cardiff University, Hadyn Ellis Building, Maindy Road, CF24 4HQ Cardiff, UK.; 7 Department of Molecular Mechanisms of Disease, University of Zurich, 8057 Zurich, Switzerland.; 8 Institute for Genetics and Genomics in Geneva (iGE3), University of Geneva, 1211 Geneva, Switzerland.

## Abstract

Expanded CAG/CTG repeats underlie 13 neurological disorders, including myotonic dystrophy type 1 (DM1) and Huntington’s disease (HD). Upon expansion, disease loci acquire heterochromatic characteristics, which may provoke changes to chromatin conformation and thereby affect both gene expression and repeat instability. Here, we tested this hypothesis by performing 4C sequencing at the *DMPK* and *HTT* loci from DM1 and HD–derived cells. We find that allele sizes ranging from 15 to 1700 repeats displayed similar chromatin interaction profiles. This was true for both loci and for alleles with different DNA methylation levels and CTCF binding. Moreover, the ectopic insertion of an expanded CAG repeat tract did not change the conformation of the surrounding chromatin. We conclude that CAG/CTG repeat expansions are not enough to alter chromatin conformation in cis. Therefore, it is unlikely that changes in chromatin interactions drive repeat instability or changes in gene expression in these disorders.

## Teaser

Unexpectedly, the molecular pathogenesis of expanded CAG/CTG diseases does not include changes in 3D chromatin conformation.

## INTRODUCTION

The genome is organized into hierarchical chromatin contact domains (*1*). This three-dimensional (3D) organization of chromatin in the nucleus has a profound impact on transcription, DNA replication, recombination, and repair (*1*, *2*). For instance, heterochromatic and euchromatic loci are spatially separated and display distinct 3D chromatin interactions as gleaned by microscopy and chromosome conformation capture (3C)–based experiments (*2*, *3*). However, how this higher-order chromatin structure impinges on biological functions, what determines chromatin domain boundaries, and how it contributes to disease is unclear (*4*). Expanded CAG/CTG repeat loci are ideal to address these questions because they are disease-associated loci with changes in local chromatin structure, transcriptional output, and genetic instability (*5*).

CAG/CTG repeats underlie 13 different neurological and neuromuscular disorders including myotonic dystrophy type 1 (DM1) and Huntington’s disease (HD). They are part of a larger group of diseases caused by the expansion of short tandem repeats (STRs) (*6*). Disease-associated STRs (daSTRs) are genetically unstable, especially once they surpass a critical threshold of about 35 to 50 repeat units. Their expansion is also associated with extensive chromatin remodeling of the expanded loci (*5*, *7*). Two examples of diseases that are accompanied by extensive changes in chromatin marks are fragile X syndrome (FXS), caused by the expansion of CGG repeats in the *FMR1* gene located on the X chromosome (*8*–*10*), and Friedreich’s ataxia (FRDA), caused by a homozygous GAA repeat expansion in the first intron of the *FXN* gene (*11*). In the case of FXS, expansions beyond 200 CGGs are associated with promoter silencing in cis. This locus accumulates high levels of heterochromatic marks including CpG methylation, H4K20me3, H3K9me2/3, and H3K27me3 while losing euchromatin-associated marks such as H3 and H4 acetylation as well as H3K4me2 (*12*–*16*). Similar chromatin remodeling occurs at the *FXN* locus in FRDA patient tissues where expanded *FXN* alleles display increased DNA methylation, H3K9me2/3, H3K27me3, HP1 recruitment, and a loss of CCCTC-binding factor (CTCF) binding at sites flanking the expanded GAA repeats (*17*–*21*).

The shift from a euchromatic to a heterochromatic state upon daSTR expansion has led to the hypothesis that there is a concurrent change in the higher-order chromatin conformation of the surrounding genomic region. 3C-based experiments revealed alterations in 3D chromatin interactions surrounding expanded GAA and CGG repeats in patient-derived lymphoblastoid cell lines (LCLs) (*13*, *17*, *22*). In FRDA, a 3C anchor in exon 1 of *FXN* showed significantly higher interactions with genomic sites up to 39 kb upstream and 45 kb downstream of the GAA repeats in FRDA patient-derived LCLs compared to unaffected cells (*17*). In FXS patient postmortem brain tissue, fibroblasts, and LCLs, CGG repeat expansions were associated with the disruption of a topologically associating domain (TAD) boundary near the *FMR1* gene, decreased interactions near the repeat tract, and increased chromatin interactions within the upstream chromatin domain (*13*, *22*). Furthermore, daSTRs were found predominantly at TAD and sub-TAD boundaries enriched in CpG islands, suggesting more generally that daSTR expansions may disrupt TADs (*22*). Thus, it was speculated that altered chromatin conformation near expanded daSTR loci might contribute to repeat instability, transcriptional misregulation in cis, and ultimately to disease progression (*13*, *17*, *22*).

Two critical unknowns in this model are whether changes in higher-order chromatin structure are confined to CGG and GAA repeats or if this is general to daSTRs and whether changes in chromatin structure cause alterations in gene expression and repeat instability. The latter question is especially appealing in the context of the expanded CTG repeats in the 3′ untranslated region of the *DMPK* gene in DM1. Similar to *FMR1* and *FXN*, this locus undergoes heterochromatinization upon repeat expansion. The changes in local chromatin marks observed at the *DMPK* locus include the loss of CTCF binding, loss of a DNase I hypersensitive site, an increase in DNA and H3K9 methylation, as well as a loss of histone acetylation around the repeat tract (*23*–*27*). Moreover, the ectopic introduction of an expanded CAG repeat locus in budding yeast was sufficient to relocate the locus to the nuclear periphery in S phase cells, leading to changes in repeat size (*28*). Expanded CAG/CTG repeats cause most daSTR disorders. Together, these observations prompted us to test the hypothesis that chromatin conformation is altered at expanded CAG/CTG repeat loci. We used 4C sequencing (4C-seq) to determine the 3D chromatin interactions established around the *FMR1*, *HTT*, and *DMPK* loci in unaffected and FXS, HD, and DM1 patient LCLs. We also analyzed the chromatin interactions of an ectopic CAG repeat expansion in human embryonic kidney (HEK) 293–derived cells. We confirmed the alteration of chromatin interactions in a FXS patient cell line with ~935 CGG repeats. However, we found no evidence of changes in chromatin interactions caused by expanded CAG/CTG repeats in HD or DM1 patient cells. This suggests that 3D conformational changes are unlikely to underlie the alterations in transcriptional output or genetic instability of disease-associated expanded CAG/CTG repeat loci. This was consistent in different cell types, genetic backgrounds, and in the presence of specific heterochromatic marks. Therefore, we conclude that changes in higher-order chromatin conformation are unlikely to contribute to the pathogenesis of expanded CAG/CTG repeat disorders.

## RESULTS

### Chromatin conformation changes upon CGG repeat expansion at the *FMR1* locus

**Fig. 1:**
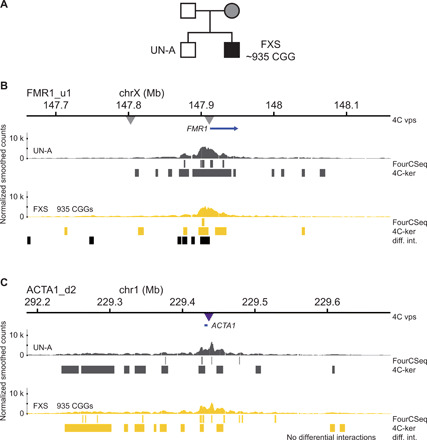
Chromatin interactions of the *FMR1* locus in unaffected and FXS patient cells. (**A**) Pedigree of the unaffected and FXS patient cell lines used. (**B**) 4C-seq chromatin interaction profiles (average of triplicate smoothed and normalized counts) from the FMR1_u1 viewpoint (1 kb upstream of the CGG repeats of *FMR1*, gray central triangle) in one unaffected (UN-A) and one FXS patient cell lines (FXS). The top blue arrow represents the *FMR1* gene and the left-side triangle represents the location of the FMR1_u195 4C viewpoint. The interaction profiles for the FMR1_u195 viewpoint (195 kb upstream of the CGG repeats of *FMR1*) can be found in fig. S2. (**C**) 4C-seq chromatin interaction profiles (average of triplicate smoothed and normalized counts) from the *ACTA1* viewpoint (central purple triangle). The top blue bar represents the *ACTA1* gene. For (B) and (C), high-interacting regions were called using 4C-ker and significant interactions were called using FourCSeq. Regions of differential interactions compared to UN-A are marked with black bars below each 4C-seq track and labeled as “diff. int.”. 4C vps, viewpoints used for 4C-seq.

To determine the chromatin conformation of expanded daSTRs, we used 4C-seq (*29*) to maximize the resolution of chromatin interactions at these loci. This method allows a high sensitivity to small changes in conformation that may be missed by other 3C-based methods. To test our approach, we sought to reproduce the observation of changes in chromatin conformation at the *FMR1* locus upon CGG repeat expansion with 4C-seq (*13*, *22*). We used two LCLs that were used in a previous study (*22*), one derived from a patient with FXS with ~935 CGG repeats (GM09237) and the other derived from their unaffected male sibling (GM09236), here referred to as FXS and UN-A, respectively (Fig. 1A and table S1).

To determine the chromatin interactions established at the *FMR1* locus, we used a 4C viewpoint located 1 kb upstream of the CGG repeats and another at 195 kb upstream (table S2). We also determined the chromatin conformation of an unrelated locus, *ACTA1* on chromosome 1, to control for a potential effect of FXS on genome-wide chromatin conformation. We obtained three replicates of each 4C viewpoint and found that replicates from the same cell line showed good correlation in 4C fragments with at least 20 mapped reads (fig. S1, A to C). We identified interaction peaks and broader high-interacting regions that contacted the 4C viewpoints at frequencies higher than expected given their linear distance away from the viewpoints. These significant interactions were determined using two 4C-seq data analysis packages: FourCSeq (*30*) and 4C-ker (*31*). We then compared the chromatin interaction profiles between the UN-A and FXS cell lines to identify regions of significant differential interaction frequency (called differential interactions; see Materials and Methods) (Fig. 1, B and C). As expected from 5C interaction maps (*22*), we found significant differences in chromatin interactions near the CGG repeats of *FMR1*, where the FXS patient cells showed decreased interactions encompassing the expanded repeats and the upstream regions of *FMR1* (Fig. 1B). By contrast, we did not find significant changes in chromatin interactions with a viewpoint located 195 kb upstream of *FMR1*, located within the upstream TAD (fig. S2). In addition, at the *ACTA1* locus, we found that the interaction profiles were similar between the two cell lines within a 2-Mb region around the viewpoint (Fig. 1C). This suggests that CGG repeat expansion alters chromatin interactions only in the vicinity of the *FMR1* locus. Thus, we confirmed previous observations made with 5C and we concluded that changes in chromatin conformation caused by daSTRs could readily be detected with 4C-seq.

### Chromatin conformation is stable upon CAG repeat expansion at the *HTT* locus

**Fig. 2:**
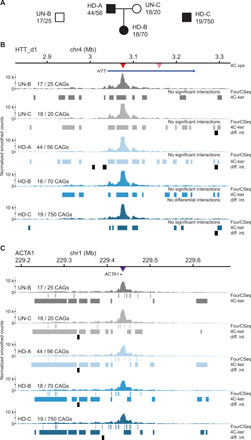
Chromatin interactions of the *HTT* locus in unaffected and HD patient cells. (**A**) Pedigree of the unaffected and HD patient cell lines used. (**B**) 4C-seq chromatin interaction profiles (average of triplicate smoothed and normalized counts) from the HTT_d1 viewpoint (1 kb downstream of the CAG repeats of *HTT*, red central triangle) in two unaffected (UN-B and UN-C) and three HD patient cell lines (HD-A, HD-B, and HD-C). The top blue arrow represents the *HTT* gene and the triangles represent the location of both *HTT* viewpoints. The interaction profiles for the HTT_d85 viewpoint (85 kb downstream of the CAG repeats) can be found in fig. S4. (**C**) 4C-seq chromatin interaction profiles (average of triplicate smoothed and normalized counts) from the *ACTA1* viewpoint (central purple triangle). The top blue bar represents the *ACTA1* gene. For (B) and (C), high-interacting regions were called using 4C-ker and significant interactions were called using FourCSeq. Regions of differential interactions compared to UN-B are marked with black bars below each 4C-seq track and labeled as “diff. int.”.

To assess whether chromatin interactions change upon CAG repeat expansion at the *HTT* locus, we used three HD patient–derived LCLs: GM02164 (44 and 56 CAGs), GM03620 (18 and 70 CAGs), and GM14044 (19 and 750 CAGs), referred to as HD-A, HD-B, and HD-C, respectively. We compared them to two cell lines from unaffected individuals (GM04604 and GM02180, UN-B and UN-C, respectively). Their family relationships and their repeat sizes are shown in Fig. 2A and table S1.

To determine the chromatin conformation of the *HTT* locus, we used two 4C viewpoints within the *HTT* gene body—1 kb (HTT_d1) and 85 kb (HTT_d85) downstream of the CAG repeats in unaffected and HD patient cells (table S2). We also assessed the chromatin conformation of the unrelated *ACTA1* gene. Replicates from the same cell lines showed good correlation in fragments with more than 20 mapped reads (fig. S1, D to F). Compared to the UN-B and UN-C cells, we found that the chromatin interaction profiles were similar within a 2-Mb region around the *HTT* viewpoints in all three HD patient LCLs (Fig. 2B and fig. S4). The *ACTA1* viewpoint also produced indistinguishable interaction profiles between unaffected and HD patient cells (Fig. 2C). We identified a few small regions displaying differential interactions, but they were mainly outside regions of significant interactions (Fig. 2, B and C). In addition, most regions of differential interactions were not exclusive to HD patient cells, as they were also found in comparisons between the two unaffected cell lines (Fig. 2, B and C). This suggests that the minor changes in chromatin interaction frequencies are due to factors other than the presence of expanded CAG repeats in *HTT*, for example, a difference in genetic background. Together, our results show that expanded CAG repeats at the *HTT* locus do not cause significant alterations of its chromatin conformation.

To determine the relationship, if any, of chromatin conformation at the *HTT* locus with gene expression in cis, we performed RNA sequencing (RNA-seq) experiments with the same unaffected and HD patient cells. Differential gene expression analysis between the HD and unaffected LCLs identified 1183 significantly up-regulated and 1307 significantly down-regulated genes (log_2_ fold change > 0.5, adjusted *P* < 0.05; fig. S5A and table S3). Within a 2-Mb region centered around *HTT*, we identified 4 genes of 25 that showed significant differential expression between the HD patient and the unaffected cell lines (fig. S5A). This proportion was not statistically different from the proportion of differentially expressed genes in a 2-Mb region around *ACTA1* (Fisher’s exact test, *P* = 0.12). Given that the chromatin interactions did not significantly change between the HD and unaffected cell lines in the *HTT* region, our data suggest that changes in gene expression in cis occur independently of the chromatin conformation of expanded *HTT* alleles in HD patient cells.

### Chromatin conformation is stable upon CTG repeat expansion at the *DMPK* locus

**Fig. 3:**
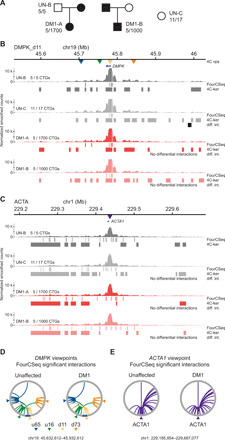
Chromatin interactions of the *DMPK* locus in unaffected and DM1 patient cells. (**A**) Pedigree of the unaffected and DM1 patient cell lines used. (**B**) 4C-seq chromatin interaction profiles (average of triplicate smoothed and normalized counts) from the DMPK_d11 viewpoint (11 kb downstream of the CTG repeats of *DMPK*, yellow triangle) in two unaffected (UN-B and UN-C) and two DM1 patient cell lines (DM1-A and DM1-B). The top blue arrow represents the *DMPK* gene and the triangles represent the location of the four *DMPK* viewpoints. The profiles for the three other viewpoints can be found in fig. S7. (**C**) 4C-seq chromatin interaction profiles (average of triplicate smoothed and normalized counts) from the *ACTA1* viewpoint (central purple triangle) in two unaffected and two DM1 patient cell lines. The top blue bar represents the *ACTA1* gene. For (B) and (C), high-interacting regions were called using 4C-ker and significant interactions were called using FourCSeq. Regions of differential interactions compared to UN-B are marked with black bars below each 4C-seq track and labeled as “diff. int.”. (**D**) Circos plot of the significant interactions called with FourCSeq (nominal *P* < 0.05) from four different viewpoints surrounding the CTG repeats of *DMPK* in the unaffected and DM1 cell lines (left and right, respectively) (DMPK_u65 in blue, DMPK_u16 in green, DMPK_d11 in yellow, and DMPK_d73 in orange; 65 kb upstream, 16 kb upstream, 11 kb downstream, and 73 kb downstream, respectively). (**E**) Circos plot of the significant interactions called with FourCSeq (nominal *P* < 0.05) from the control *ACTA1* viewpoint in unaffected and DM1 cell lines (left and right, respectively).

Repeat expansion at the *HTT* locus is not known to be associated with changes in local histone modifications and chromatin accessibility, which might cause changes in chromatin conformation. To determine whether an expanded CTG repeat locus with local heterochromatic chromatin marks is associated with altered 3D chromatin conformation, we used the unaffected cell lines UN-B and UN-C and two DM1 patient–derived LCLs (GM06077 and GM04648, referred to as DM1-A and DM1-B, respectively). The DM1-A cell line harbored one expanded *DMPK* allele with 1700 CTGs and the DM1-B cell line, 1000 CTGs (Fig. 3A and table S1). Using bisulfite sequencing, we found that DM1-A cells harbored increased CpG methylation levels at two CTCF binding sites flanking the CTG repeats (fig. S6, A and B). To assess the CTCF occupancy levels at the two flanking sites, we performed CTCF chromatin immunoprecipitation (ChIP) followed by quantitative PCR (qPCR) and found that CTCF binding was reduced at both sites in the DM1-A cell line (fig. S6C). In DM1-B cells, we observed normal methylation levels at both CTCF binding sites (fig. S6B) and slightly reduced CTCF occupancy in the downstream site (fig. S6C). Thus, DM1-A cells displayed molecular signatures of congenital DM1 (*32*), whereas DM1-B cells had characteristics of adult-onset DM1 (table S1).

To determine whether expanded CTG repeats affect chromatin conformation at the *DMPK* locus, we performed 4C-seq with the unaffected and DM1 cell lines using four different 4C viewpoints at distinct distances away from the CTG repeats of *DMPK* (Fig. 3B, fig. S7, and table S2). Replicates from the same cell lines also showed good correlation for 4C fragments with at least 20 reads (fig. S1, G to K). Similar to the HD samples, we observed notably similar chromatin interaction profiles between the unaffected and DM1 samples for all four viewpoints in the *DMPK* region (Fig. 3B and fig. S7). As expected, the *ACTA1* viewpoint also showed interaction profiles indistinguishable between unaffected and DM1 cell lines (Fig. 3C). As with the *HTT* viewpoints, none of the *DMPK* or *ACTA1* viewpoints in DM1 patient cells had significant interactions that were also called as regions of differential interaction (Fig. 3, B and C). Furthermore, we found that the significant interactions identified for all four *DMPK* viewpoints and the *ACTA1* viewpoint in unaffected and DM1 patient cells were largely the same (Fig. 3, D and E). Thus, we found no evidence of large-scale changes in chromatin conformation at the *DMPK* locus driven by CTG expansions.

We confirmed by RNA-seq that transcriptional misregulation in DM1 patient cells occurs genome-wide as well as within a 2-Mb region centered around *DMPK* (fig. S5B and table S4). Similar to our observation in HD individuals, changes in gene expression in cis occurred independently of the 3D conformation of the *DMPK* locus in DM1 patient cells.

To determine the extent of CTCF occupancy alterations in the DM1 patient cells, we performed ChIP sequencing (ChIP-seq) (fig. S8). At the genome-wide level, comparing DM1-A to UN-B cells, we found 1951 sites where CTCF binding changed significantly [false discovery rate (FDR) < 5%]. DM1-B cells harbored 833 sites with significantly altered CTCF occupancy genome-wide compared to UN-B cells (FDR < 5%). However, in a 2-Mb region centered around *DMPK*, only 4 of 131 (3%) CTCF binding peaks showed significant differential occupancy in DM1-A patient cells compared to the UN-B cell line (fig. S8). There were no binding sites with significant differences in CTCF occupancy out of 135 peaks in that same region when comparing the UN-B and DM1-B cell lines (fig. S8). Therefore, we concluded that there were no large-scale changes in CTCF occupancy in the DM1 patient LCLs either genome-wide or within a 2-Mb region around the expanded CTG repeats, which is consistent with the absence of changes in chromatin conformation observed by 4C-seq in these cells.

### Lack of allelic bias in chromatin interactions at expanded CAG/CTG repeats

DM1 and HD are both dominantly inherited disorders wherein affected individuals are heterozygous for the expanded allele. Thus, one potential caveat in our data was that the presence of a normal-length allele could mask changes in the 3D chromatin interactions made by the expanded allele. To evaluate this possibility, we took advantage of the presence of at least one parental cell line for the DM1-A and HD-B individuals in our dataset (Figs. 2A and 3A) and identified biallelic single-nucleotide polymorphisms (SNPs) in the contacted 4C fragments from these samples. We selected a subset of the SNPs that could be assigned unambiguously to either the expanded or the normal allele within 1 Mb of the 4C viewpoints (see Materials and Methods). We reasoned that if chromatin contacts were established without a systematic bias for either the expanded or normal allele in DM1 and HD patient cells, then the proportion of 4C fragments in which the expanded chromosome had more reads than the normal-length one would be close to 50%. Therefore, we analyzed the sequencing coverage of the 4C-seq data at the biallelic SNP positions and found that the viewpoints did not establish preferential contacts with a single chromosome in either the DM1-A or HD-B patient cell lines (Table 1). These results are consistent with the conclusion that chromatin interactions at both disease loci do not show allelic bias. Together, these results corroborate the conclusion that expanded CAG/CTG repeats do not significantly alter the chromatin interactions at two expanded CAG/CTG repeat loci.

**Table 1:** Allele-specific interactions in 4C-seq data.

**Cell line**	**Disease**	Genomic region*	**Total no. of** **samples**	**No. of samples** **with more normal** **chromosome** **reads**	**No. of samples** **with more** **expanded** **chromosome** **reads**	***P*^†^**
DM1-A	DM1	*ACTA1*	5	1	4	0.38
		*DMPK*	41^‡^	26	15	0.12
HD-B	HD	*ACTA1*	12	7	5	0.77
		*HTT*	43	20	23	0.76

*One-megabase region around the 3′ end of *DMPK*, 5′ end of *HTT*, or the *ACTA1* 4C-seq viewpoint.

†*P* value of an exact binomial test.

‡One sample had the same number of reads in both alleles; thus, it was not included.

### Chromatin conformation is stable at an ectopic CAG repeat locus

**Fig. 4:**
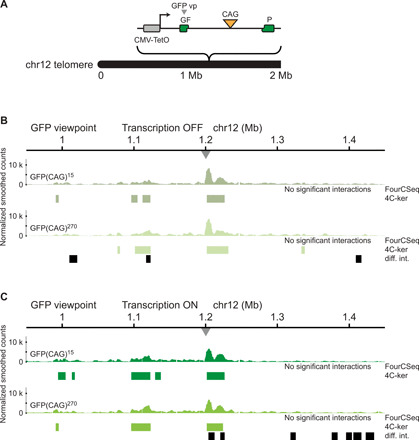
Chromatin interactions of an expanded CAG repeat locus in isogenic cells. (**A**) Diagram of the integration site of the CAG ectopic locus in GFP(CAG)_15_ and GFP(CAG)_270_ cells. (**B**) 4C-seq chromatin interaction profiles (average of triplicate smoothed and normalized counts) from the GFP viewpoint in GFP(CAG)_15_ (top) and GFP(CAG)_270_ (bottom) in cells without active transcription of the ectopic CAG locus. Regions of differential interactions compared to GFP(CAG)_15_ are marked with black bars below the GFP(CAG)_270_ 4C-seq track. (**C**) 4C-seq chromatin interactions from the GFP viewpoint in GFP(CAG)_15_ (top) and GFP(CAG)_270_ (bottom) in cells treated with doxycycline for 5 days to induce transcription of the ectopic CAG locus. Regions of differential interactions compared to GFP(CAG)_15_ are marked with black bars below the GFP(CAG)_270_ 4C-seq track.

It remained possible that differences in genetic background in the LCLs that we analyzed could have had a confounding effect on the chromatin interactions made at expanded CAG/CTG repeat loci. To test this, we compared the chromatin interactions of a hemizygous ectopic locus with either 15 [GFP(CAG)_15_] or 270 CAGs [GFP(CAG)_270_] in isogenic cell lines. We obtained two clonal populations of HEK293 T-Rex Flp-In cells that contain a single, stably integrated construct containing CAG repeats within the intron of a green fluorescent protein (GFP) mini-gene controlled by a doxycycline-inducible promoter (*33*). Using targeted locus amplification (*34*), we mapped its insertion site to the p-arm of chromosome 12, 1.2 Mb from the telomere (Fig. 4A). We performed 4C-seq in both cell lines with a viewpoint located 1 kb upstream of the CAG repeats. The chromatin interaction profiles of this ectopic CAG repeat locus were similar between the 15 and 270 CAG repeat cells, with few regions of differential interactions overlapping with high-interacting regions identified with 4C-ker (Fig. 4B). Thus, we concluded that expanded CAG repeats cause few changes to the chromatin conformation of an ectopic locus in isogenic cells.

Some studies suggest that transcription can help define chromatin domain boundaries (*35*). To determine whether transcription through expanded CAG repeats could lead to changes in chromosome conformation, we induced transcription at this locus by culturing the GFP(CAG)_15_ and GFP(CAG)_270_ cells with doxycycline for 5 days and subsequently performed 4C-seq using the same GFP viewpoint. We observed that 2 of 17 regions of differential interactions overlapped fully or in part with high-interacting regions over a 2-Mb region (Fig. 4C). By contrast, we identified no significant interactions in this region using FourCSeq. Together, these results suggest that an ectopic expanded CAG repeat tract is not enough to significantly alter the chromosome conformation of this locus, regardless of its transcriptional status.

## DISCUSSION

Here, we showed that chromatin interactions remain stable at expanded CAG/CTG repeat loci. This was true for two disease loci (*HTT* and *DMPK*) and one ectopic locus in two different cell types. Our findings are supported by allele-specific analysis of 4C-seq chromatin interactions. Furthermore, increased CpG methylation and CTCF binding alterations at four sites in a 2-Mb region around *DMPK* did not affect the chromosome conformation of this locus in the DM1-A patient cells. This is especially relevant because CTCF is a key architectural protein involved in the demarcation of TAD and sub-TAD boundaries (*36*). In addition, when we inserted a hemizygous transgene with CAG repeats, we found that expanded CAG repeats did not induce a significant reorganization of the chromatin contacts established at this ectopic repeat locus. These results show that an expanded CAG/CTG repeat tract is not sufficient to change chromatin conformation in cis.

One possible explanation for why we observed strikingly similar chromatin interactions in the expanded CAG/CTG repeat loci tested here is that changes in chromatin interactions caused by expanded daSTRs only occur in affected cell types, such as cardiomyocytes or medium spiny neurons, but not in LCLs. Such a scenario would require cell type–specific factors to mediate these changes. Here, we tested the transcriptional status, DNA methylation levels, and CTCF binding in DM1 patient LCLs. We found that these cells displayed transcriptional and epigenetic signatures similar to those found in heart, liver, cortex, and peripheral blood samples of patients with DM1 (*23*, *32*). Thus, cell type–specific differences in chromatin conformation are possible but unlikely given the similarity in local chromatin modifications between the tissues.

Our findings are in contrast to the effect of expanded CGG and GAA repeats on chromosome conformation (*13*, *17*, *22*). In FXS patient–derived LCLs, fibroblasts, and brain tissue, expanded CGG repeats in *FMR1* were associated with heterochromatic characteristics, decreased CTCF binding, and a disruption of a TAD boundary near the expanded repeats (*22*). The DM1 patient cells used here, especially the congenital DM1 patient cell line (DM1-A), showed similar changes in chromatin modifications to FXS patient cells. And yet, these factors did not amount to an alteration of the 3D chromatin interactions at the expanded *DMPK* locus. There are notable dissimilarities between FXS/FRDA and DM1/HD that could account for the difference in the effect of distinct daSTRs on chromosome conformation. Apart from the nucleotide composition of the repeat tract itself, the disease loci and their flanking sequences are different. It is possible that the extent of heterochromatin formation is different between distinct expanded repeat loci, thereby eliciting different effects on the surrounding 3D chromatin conformation. Similarly, how far CpG methylation spreads and how much CTCF occupancy is disrupted upon repeat expansion is not fully understood and may account for the differences between the daSTR loci examined so far. It seems likely that all these dissimilarities between the loci examined so far contribute to the changes, or lack thereof, in chromatin conformation upon expansion of daSTRs.

It was speculated that the changes in chromatin conformation caused by expanded daSTRs could lead to repeat instability (*22*). CGG repeat expansions beyond 200 units are associated with reduced instability, promoter silencing, and 3D chromatin conformation changes (*22*, *37*, *38*). By contrast, long GAA repeat tracts are more unstable (*6*, *39*), and yet, their chromosome conformation is also altered. We found that long CAG/CTG repeats, which are more unstable as they expand, are not associated with changes in 3D chromatin interactions. Thus, there does not appear to be a general functional link between chromatin conformation and repeat instability across distinct daSTR loci.

Similarly, we found that transcription around the *HTT* and *DMPK* regions in patients with HD and DM1 is unrelated to the chromatin conformation of the expanded repeat loci. Rather, it is more likely that the local transcriptional misregulation observed in patients is caused by changes in local chromatin marks. Our results are in line with recent evidence in *Drosophila* arguing that genome topology is not predictive of genome-wide transcriptional output (*40*). We did not find evidence that supports a common link between 3D genome organization and the transcriptional misregulation typical of expanded daSTRs. Overall, our data argue that changes in chromatin topology are unlikely to underpin the molecular pathology of expanded CAG/CTG repeat disorders.

The expanded CAG/CTG repeat loci in DM1 and HD provide an endogenous genomic substrate to study the mechanisms necessary for the establishment of 3D chromatin domains in daSTRs given the involvement of CTCF binding, CpG methylation, and other chromatin remodeling events at these loci. In particular, CTCF plays a central role in the establishment of chromatin loops and chromatin contact domains (*36*). Our data show that chromatin interactions remain stable near the *DMPK* region despite precise alterations in CTCF binding near a chromatin contact domain boundary. This is in line with several recent observations wherein interfering with CTCF binding at chromatin domain boundaries does not result in a reorganization of the underlying 3D topological chromatin structure. For example, neither the deletion nor the ectopic insertion of the *Firre* locus, a long noncoding RNA on the X chromosome surrounded by 15 CTCF binding sites, was enough to alter the TAD structure of the surrounding genomic region in mouse cells (*41*). Similarly, a TAD boundary in the *HoxD* gene cluster was highly resilient to genomic deletions encompassing multiple CTCF binding sites (*42*). Therefore, these results, together with ours, imply a model whereby the establishment and maintenance of chromatin conformation is dependent on the chromosomal context of a given genomic locus.

## MATERIALS AND METHODS

### Cell lines

All LCLs were obtained from the Coriell Institute for Medical Research Cell Repository. They were grown at 37°C and 5% CO_2_ in RPMI 1640 medium supplemented with 15% fetal bovine serum, 2 mM l-glutamine, and 1% penicillin-streptomycin. Cells were counted and passaged every 3 to 4 days depending on cell density. The GFP(CAG)_270_ and GFP(CAG)_15_ lines were previously characterized (*33*). They were maintained at 37°C and 5% CO_2_ in Dulbecco’s minimum Eagle’s medium with GlutaMAX, 1% penicillin-streptomycin, blasticidine (15 μg ml^−1^), and hygromycin (150 μg ml^−1^). Transcription of the GFP mini-gene was activated by culturing GFP(CAG)*_n_* cells with doxycycline at a final concentration of 2 μg ml^−1^ for 5 days.

### Repeat length determination and small-pool PCR

Genomic DNA was isolated from each LCL using the NucleoSpin Tissue Kit (Macherey-Nagel). PCR products with the CAG repeats from *HTT* were produced with primers oVIN-1333 and oVIN-1334 (table S5). PCR products containing the CTG repeats from *DMPK* were amplified with primers oVIN-1252 and oVIN-1251 (table S5). For normal-length alleles, several PCRs were set up with MangoTaq (Bioline), and the products were gel-extracted and Sanger-sequenced with the same primers used for the amplification. For expanded alleles, small-pool PCRs were performed on the basis of a previously described protocol (*43*). Briefly, the same primers were used for the amplification of expanded *DMPK* and *HTT* alleles with 1 ng of genomic DNA per PCR. The products were run on an agarose gel and transferred to a nylon membrane. An oligo made up of 10 CAGs was used to obtain a radioactive probe used for the visualization of the expanded alleles. The number of repeats reported here is an estimation of the modal number of repeats.

### Bisulfite sequencing

Bisulfite sequencing was performed according to a previously described method (*32*). Bisulfite conversion was performed with the EZ DNA Methylation Kit (Zymo Research) using the standard protocol. Two hundred nanograms of genomic DNA was used for bisulfite conversion at 50°C for 12 hours. Bisulfite-converted DNA was desulfonated, eluted, and immediately used for nested and heminested PCR amplification of the upstream and downstream CTCF binding sites, respectively, using previously described primers (*32*). Fifty nanograms of bisulfite-converted DNA was used for the first PCR, and 3 μl of the products was used for the second PCR. The final amplicons were purified with the NucleoSpin PCR Clean-up Kit (Macherey-Nagel) and used for 2 × 250–base pair (bp) paired-end sequencing on Illumina MiSeq. The primers used for both rounds of PCR are found in table S5.

### Bisulfite sequencing data analysis

Sequencing reads were preprocessed using Trim Galore (github.com/FelixKrueger/TrimGalore) with the following parameters: -q 20 --length 20 –paired. Reads were aligned using QuasR (*44*) to the GRCh38 human reference DNA sequences corresponding to the amplified PCR products. DNA methylation calls for each CpG were extracted using the qMeth() function in QuasR. DNA methylation frequencies were calculated as methylated CpG reads / total number of reads covering the respective CpG × 100.

### CTCF ChIP-qPCR and ChIP-seq

ChIP was performed according to the Diagenode Auto iDeal ChIP-qPCR Kit (Diagenode) standard protocol. Samples of 6 × 10^6^ cells were sonicated using the Diagenode Bioruptor Pico (Diagenode), with 10 cycles of 30 s “on” and 30 s “off.” Correct DNA fragmentation was verified by agarose gel electrophoresis. Immunoprecipitation was performed with 4 × 10^6^ cells using a CTCF antibody (Diagenode) and the Diagenode IP-Star Compact Automated System robot (Diagenode). Results were analyzed using the StepOnePlus qPCR by Applied Biosystems (Thermo Fisher Scientific) with Applied Biosystems SYBR Green PCR Master Mix (Thermo Fisher Scientific). The primer sequences used for qPCR are listed in table S5. CTCF ChIP-seq libraries were prepared in triplicate using the Illumina TruSeq ChIP Sample Preparation Kit. We performed 1 × 50-bp single-end sequencing with Illumina HiSeq 4000.

### CTCF ChIP-seq data analysis

CTCF ChIP-seq reads were demultiplexed with the bcl2fastq2 Illumina software (v 2.20.0). Data analysis was performed according to the ENCODE Transcription Factor ChIP-seq processing pipeline (phase 3). Demultiplexed reads were aligned to the GRCh38 human reference genome (GCA_000001405.15) using bowtie2 (v 2.3.4.3) (*45*). Reads were filtered to remove unmapped reads, reads with no primary alignment, multimapped reads, and duplicate reads so that only the reads that mapped to the genome once were considered for downstream analyses. CTCF peaks were identified using the ChIP-seq processing pipeline (*46*). The irreproducible discovery rate (*47*) framework was used to ensure reproducibility between experimental replicates. Differentially enriched CTCF peaks were detected with DiffBind (*48*). We used DESeq2 (*49*) to identify peaks that are statistically differentially bound between sample groups (*P* < 0.05 and FDR < 5%). First, we computed count information for each of the peaks in the consensus set. Bedgraph files (representing a normalized coverage per base) were created to visualize the tracks.

### 4C sequencing

4C library preparation was performed on the basis of a previously described protocol (*29*). For each sample, 10^7^ cells were cross-linked in 2% formaldehyde for 10 min at room temperature and quenched with glycine to a final concentration of 0.13 M. Cross-linked samples were rinsed once with phosphate-buffered saline and were either used immediately or flash-frozen in liquid nitrogen and stored at −80°C for later use. Cells were lysed for 15 min on ice in lysis buffer [50 mM tris-HCl (pH 7.5), 150 mM NaCl, 5 mM EDTA, 0.5% NP-40, 1% Triton X-100, and 1× protease inhibitors]. The first digestion was performed with 200 U of Dpn II (New England Biolabs) and incubating for 4 hours at 37°C, then 200 U of Dpn II and overnight incubation at 37°C, and lastly 200 U of Dpn II and incubating 4 hours at 37°C. Dpn II digestion efficiency was assessed by agarose gel electrophoresis, and Dpn II was subsequently heat inactivated. The first ligation was performed at 16°C overnight with 50 U of T4 DNA ligase (Thermo Fisher Scientific) in 7 ml. Ligated samples were decross-linked with 30-μl Proteinase K (10 mg/ml) at 65°C overnight followed by ribonuclease A (RNAse A) treatment and phenol-chloroform purification. The second digestion was performed with 50 U of Bfa I (New England Biolabs) at 37°C overnight followed by Bfa I heat inactivation. The second ligation was performed at 16°C overnight with 100 U of T4 DNA ligase in 14 ml. The resulting 4C template samples were precipitated and purified with the QIAquick PCR purification Kit. 4C libraries were generated by amplifying 1-μg total of purified 4C template using the Expand Long Template PCR System (Roche) with 4C viewpoint primers with Illumina sequencing adapters that permitted multiplexing (in 50-μl PCRs), pooling reactions, and purifying the PCR products with AMPure XP beads to exclude products less than 130 bp. The 4C viewpoint primer sequences are listed in table S5. Single-end sequencing of pooled 4C libraries was performed on Illumina HiSeq 2500.

### 4C-seq data analysis

Demultiplexing, trimming, and mapping were performed using the BBCF HTSstation (*50*), according to (*51*). Reads were trimmed to keep the first 40 bp. The 4C fragments surrounding the viewpoints (±2.5 kb) were excluded from the rest of the analysis. The demultiplexed reads were mapped to the GRCh38 human reference genome using bowtie2 (v 2.2) (*45*). Fragment read counts were obtained using FourCSeq (v 1.18.0) (*30*). The number of mapped reads for each sample is found in table S2. For plotting the data, fragment counts were normalized (reads per million) and smoothed with a running mean (window size = 5 fragments). The smoothed and normalized fragment counts were averaged among replicates of the same 4C library samples and visualized with gFeatBrowser (www.gfeatbrowser.com). Significant chromatin interactions were identified with two 4C-seq data analysis packages: FourCSeq (v 1.18.0) (*30*) and 4C-ker (v 0.0.0.9000) (*31*). For the FourCSeq analysis, we defined significant interactions as fragments with a *z* score equal to or greater than 1.96 and an FDR of 0.1, using the following parameters: minCount = 20 and fitFun = “distFitMonotone” in the getZScores function; zScoreThresh = 1.96, fdrThresh = 0.1 in the addPeaks function. 4C-ker uses a Hidden Markov Model that accounts for differences in coverage near 4C viewpoints to determine three types of domains: high-interacting, low-interacting, and noninteracting domains. For each viewpoint, we used *k* = 5 in the nearBaitAnalysis function and plotted the high-interacting regions. The difference between significant interactions called with FourCSeq and 4C-ker is expected given that FourCSeq usually identifies “peaks” of significantly interacting regions whereas 4C-ker identifies regions (*52*). Differential interactions were identified with the differentialAnalysis function of 4C-ker, which is based on the DESeq2 (*49*) framework, using default parameters (including a *P* value threshold of 0.05). The UN-B cell line was used as the reference condition for all comparisons except for Fig. 1 and fig. S2 where the reference condition was the UN-A cell line.

### 4C SNP data analysis

We called SNPs from 4C-seq data from the DM1-A and HD-B patient–derived LCLs using GATK (v 3.7.0) (*53*) and samtools/bcftools (v 1.5 and v 1.4.1, respectively) (*54*). Biallelic SNPs located within a 1-Mb region of the 3′ end of *DMPK*, the 5′ end of *HTT*, and the *ACTA1* 4C viewpoint were selected. Among these, only SNPs that could unambiguously inform which parental allele they came from were retained for downstream analysis. This required a homozygous genotype in at least one of the parental cell lines. We validated a subset of these SNPs by isolating genomic DNA from the UN-B, UN-C, HD-A, HD-B, and DM1-A cell lines and using it for PCR amplification of the genomic region encompassing the variants in the parental and offspring LCLs followed by library preparation and 2 × 250-bp paired-end sequencing was performed with Illumina MiSeq. We then analyzed the sequencing coverage at the confirmed biallelic SNP positions from the 4C-seq data from DM1-A and HD-B patient cell lines. For samples with at least 10 reads per SNP position, we counted the number of times the expanded allele had more mapped reads than the normal allele. We applied an exact binomial test to statistically assess whether the proportion of cases where the expanded allele had more reads than the normal allele was significantly different to 0.5, which represented the null hypothesis of no allelic bias.

### RNA isolation and sequencing

For each sample, RNA from 5 million cells was extracted using the NucleoSpin RNA Kit (Macherey-Nagel), and its concentration was measured by NanoDrop (NanoPhotometer NP80, Implen). RNA quality was assessed on a Fragment Analyzer (Agilent Technologies), and all of the RNA samples had an RNA quality number between 8.7 and 10. RNA-seq libraries were prepared using 500 ng of total RNA and the Illumina TruSeq Stranded Total RNA Library Prep Kit with Ribo-Zero Gold (Illumina) according to the manufacturer’s protocol. Multiplexed samples were pooled in equimolar amounts, and 2 × 100-bp paired-end sequencing was performed on Illumina HiSeq 2500.

### RNA-seq data analysis

RNA-seq reads were demultiplexed using the bcl2fastq Illumina software (v 2.20.0). Purity-filtered reads were trimmed with Cutadapt (v 1.3) (*55*) and filtered for low complexity with seq_crumbs (v 0.1.8). Reads were aligned against the GRCh38 human transcriptome (GRCh38.82) using STAR (v 2.4.2a) (*56*). Genes differentially expressed in HD and DM1 LCLs compared to unaffected LCLs were determined using DESeq2 (v 1.20.0) (*49*) while accounting for batch effects including cell passage number, collection date, and sequencing runs. *P* values were adjusted using the Benjamini-Hochberg multiple testing correction. Significantly differentially expressed genes were defined as those with adjusted *P* values less than 0.05 and log_2_ fold change greater than 0.5. Functional gene ontology enrichment analyses of significantly differentially expressed genes in HD and DM1 LCLs were performed using the DAVID tool (v 6.8) (*57*, *58*), and all of the expressed genes in HD and DM1, respectively, were used as the background.

### Targeted locus amplification

Targeted locus amplification (TLA) was performed on the basis of a previously described protocol (*34*). For each sample, 10^7^ cells were cross-linked in 2% formaldehyde for 10 min at room temperature and quenched with glycine to a final concentration of 275 mM. Cell were lysed for 5 min at room temperature in lysis buffer [50 mM tris-HCl (pH 7.5), 150 mM NaCl, 5 mM EDTA, 0.5% NP-40, 1% Triton X-100, and 1× protease inhibitors). Cross-linked samples were digested at 37°C overnight with 400 U of Nla III (New England Biolabs) followed by Nla III heat inactivation. Samples were ligated at room temperature for 2 hours with 20 U of T4 DNA ligase (Thermo Fisher Scientific) in 500 μl. Ligated samples were decross-linked with 5-μl Proteinase K (10 mg/ml) at 65°C overnight, followed by RNAse A treatment and phenol-chloroform purification. Samples then digested overnight at 37°C with 50 U of Nsp I (New England Biolabs) followed by Nsp I inactivation. The second ligation was performed overnight at 16°C with 100 U of T4 DNA ligase in 14 ml. The resulting TLA circularized templates were purified with the QIAquick PCR Purification Kit. TLA libraries were generated by amplifying 800 ng of purified TLA template with TLA viewpoint primers (table S5). Paired-end sequencing (2 × 150 bp) of pooled TLA libraries was performed on Illumina HiSeq 4000. Demultiplexed reads were mapped using a custom TLA analysis pipeline using the Burrows-Wheeler Aligner mapping software (v 0.7.17) (*59*). First, reads were mapped to the human genome GRCh38. Then, unaligned sequences were digested in silico with the Nla III restriction site and remapped to the genome. The combined mapping results were used to determine the integration site.

## Supplementary Material

aaz4012_SM.pdf

aaz4012_Table_S3.xlsx

aaz4012_Table_S4.xlsx

## SUPPLEMENTARY MATERIALS

Supplementary material for this article is available at http://advances.sciencemag.org/cgi/content/full/6/27/eaaz4012/DC1

## Appendix

View/request a protocol for this paper from *Bio-protocol*.
