# Supplementary material for: Three-dimensional chromatin interactions remain stable upon CAG/CTG repeat expansion
Source: Sci Adv. 2020 Jul 3;6(27):eaaz4012. doi: 10.1126/sciadv.aaz4012 (PMC7334000; doi:10.1126/sciadv.aaz4012)
Supplement: aaz4012_SM.pdf [file aaz4012_SM.pdf]

Science Advances
AAAS

advances.sciencemag.org/cgi/content/full/6/27/eaaz4012/DC1

Supplementary Materials for

Three-dimensional chromatin interactions remain stable upon CAG/CTG repeat expansion

Gustavo A. Ruiz Buendía, Marion Leleu, Flavia Marzetta, Ludovica Vanzan, Jennifer Y. Tan, Victor Ythier, Emma L. Randall, Ana C. Marques, Tuncay Baubec, Rabih Murr, Ioannis Xenarios, Vincent Dion*

*Corresponding author. Email: dionv@cardiff.ac.uk

Published 3 July 2020, *Sci. Adv.* **6**, eaaz4012 (2020)
DOI: 10.1126/sciadv.aaz4012

The PDF file includes:

Figs. S1 to S8
Tables S1 and S5
References

Other Supplementary Material for this manuscript includes the following:

(available at advances.sciencemag.org/cgi/content/full/6/27/eaaz4012/DC1)

Tables S3 and S4

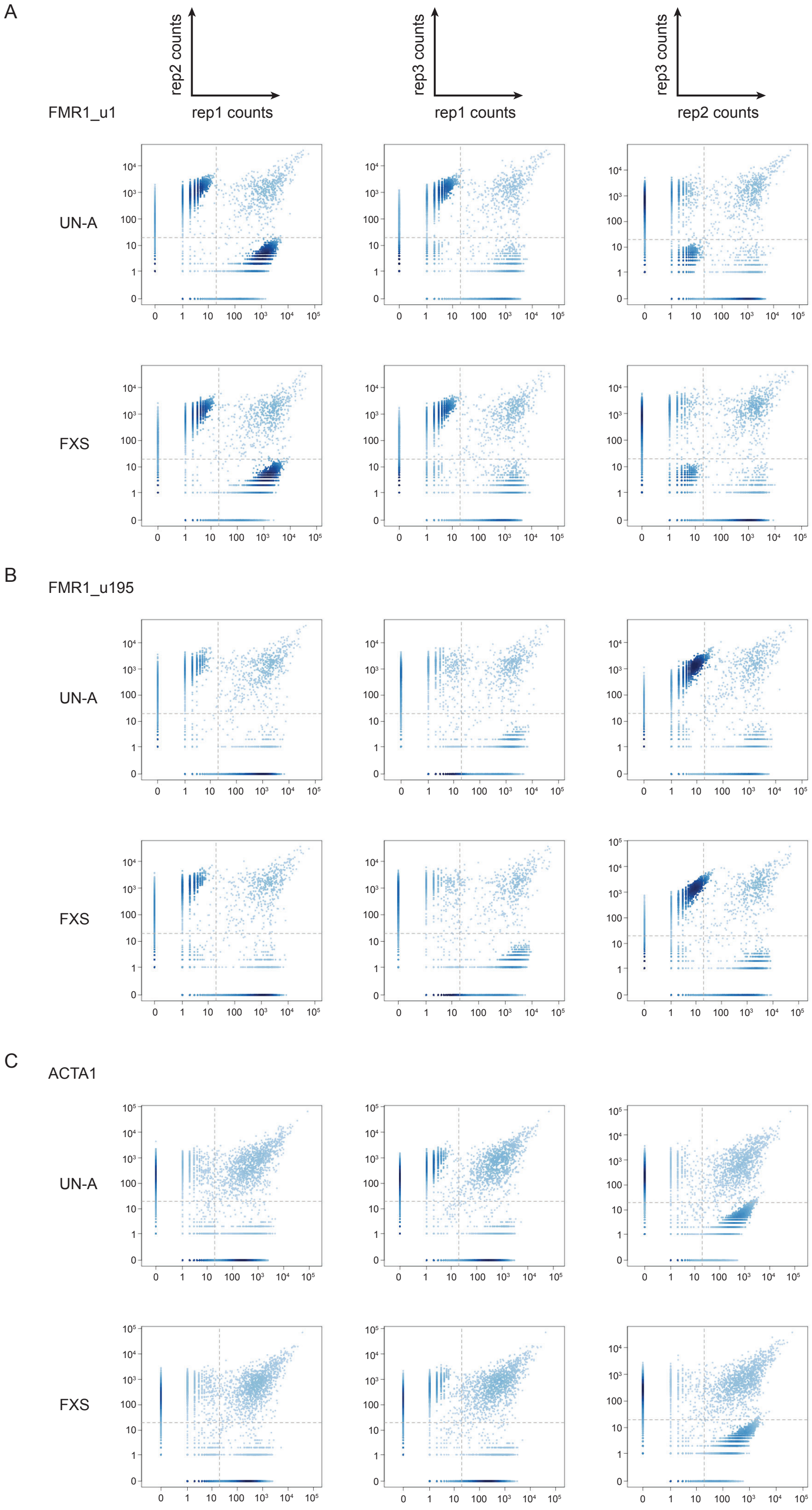
A
rep2 counts
rep1 counts
rep3 counts
rep1 counts
rep3 counts
rep2 counts
FMR1_u1
UN-A
FXS
B
FMR1_u195
UN-A
FXS
C
ACTA1
UN-A
FXS

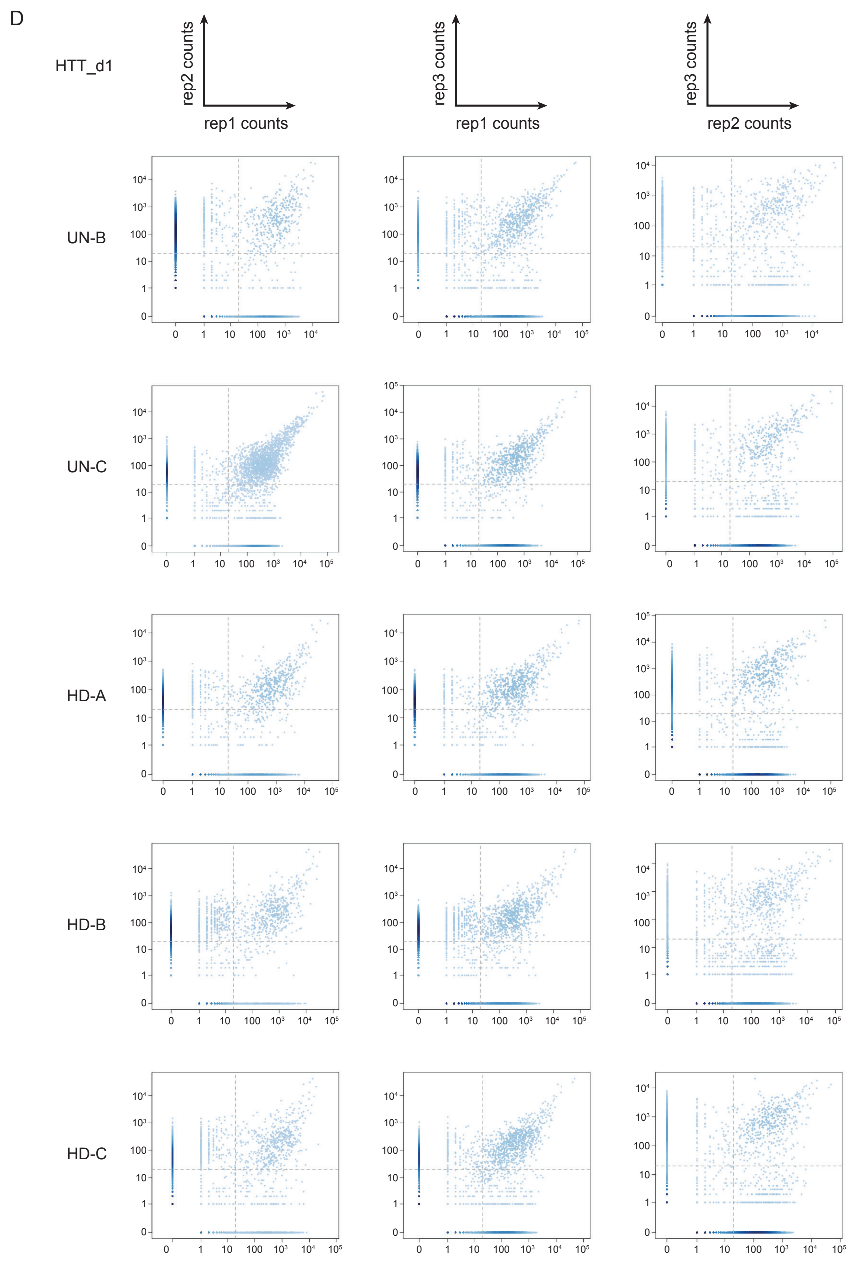
D
HTT_d1
rep2 counts
rep1 counts
rep3 counts
rep1 counts
rep3 counts
rep2 counts
UN-B
UN-C
HD-A
HD-B
HD-C

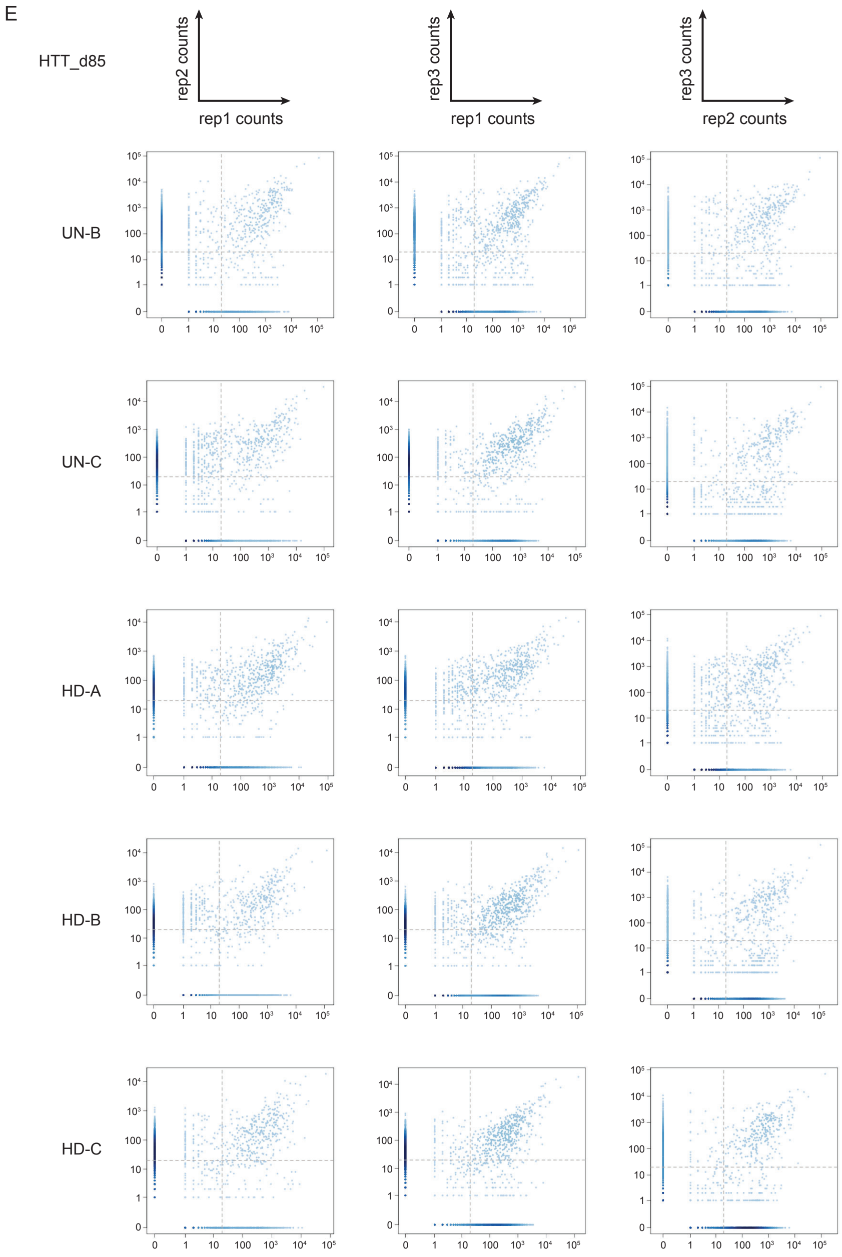
E
HTT_d85
rep2 counts
rep1 counts
rep3 counts
rep1 counts
rep3 counts
rep2 counts
UN-B
UN-C
HD-A
HD-B
HD-C

F

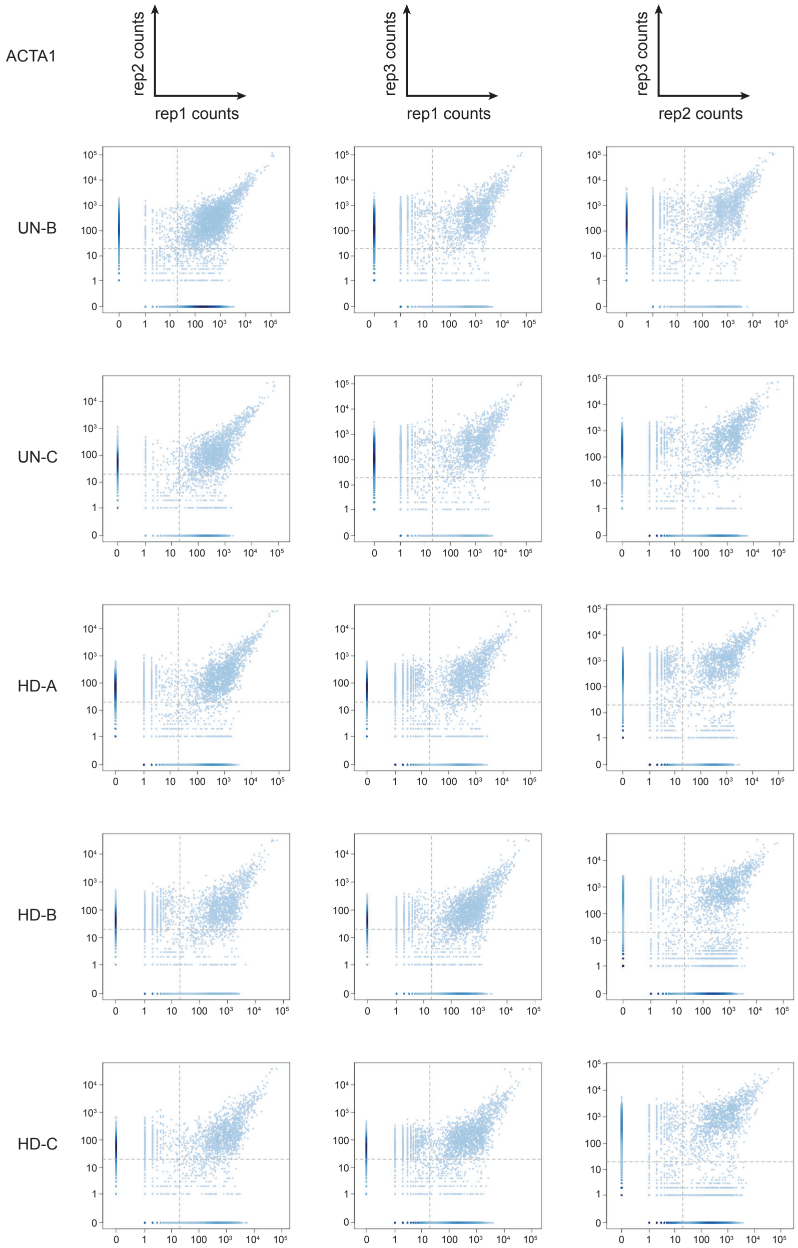
ACTA1
rep2 counts
rep1 counts
rep3 counts
rep1 counts
rep3 counts
rep2 counts
UN-B
UN-C
HD-A
HD-B
HD-C
0
1
10
100
10³
10⁴
10⁵

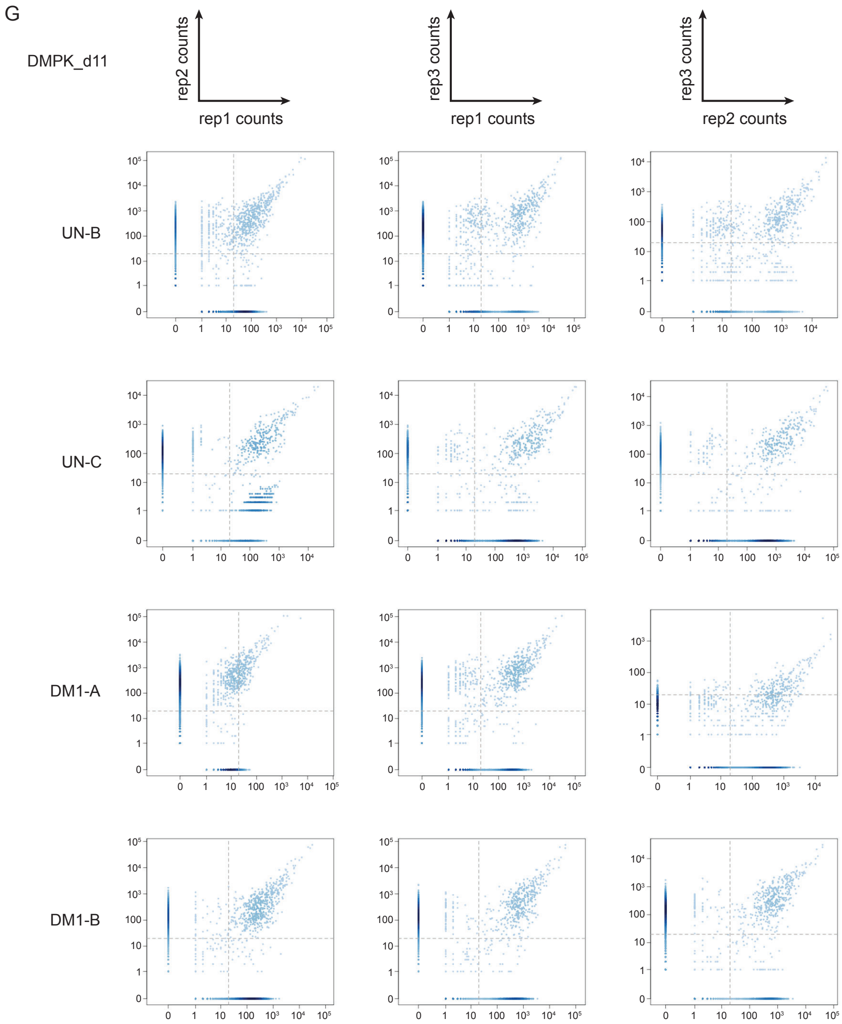
G
DMPK_d11
rep2 counts
rep1 counts
rep3 counts
rep1 counts
rep3 counts
rep2 counts
UN-B
UN-C
DM1-A
DM1-B

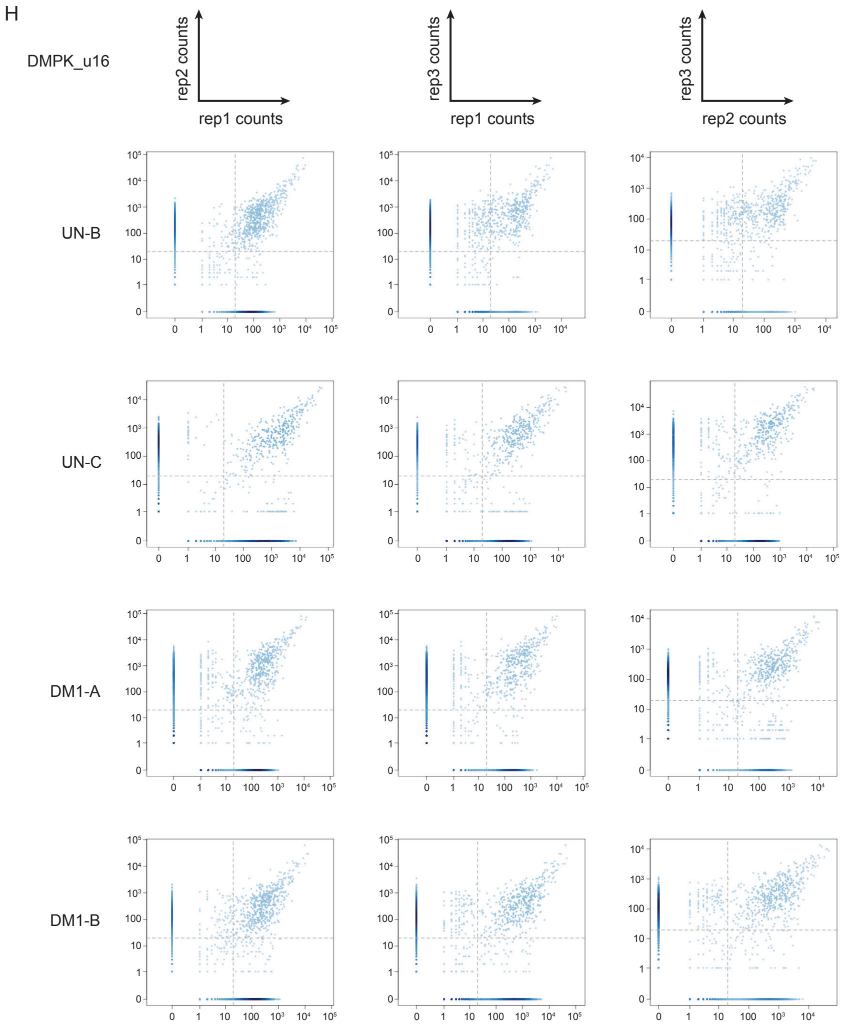
H
DMPK_u16
rep2 counts
rep1 counts
rep3 counts
rep1 counts
rep3 counts
rep2 counts
UN-B
UN-C
DM1-A
DM1-B

I

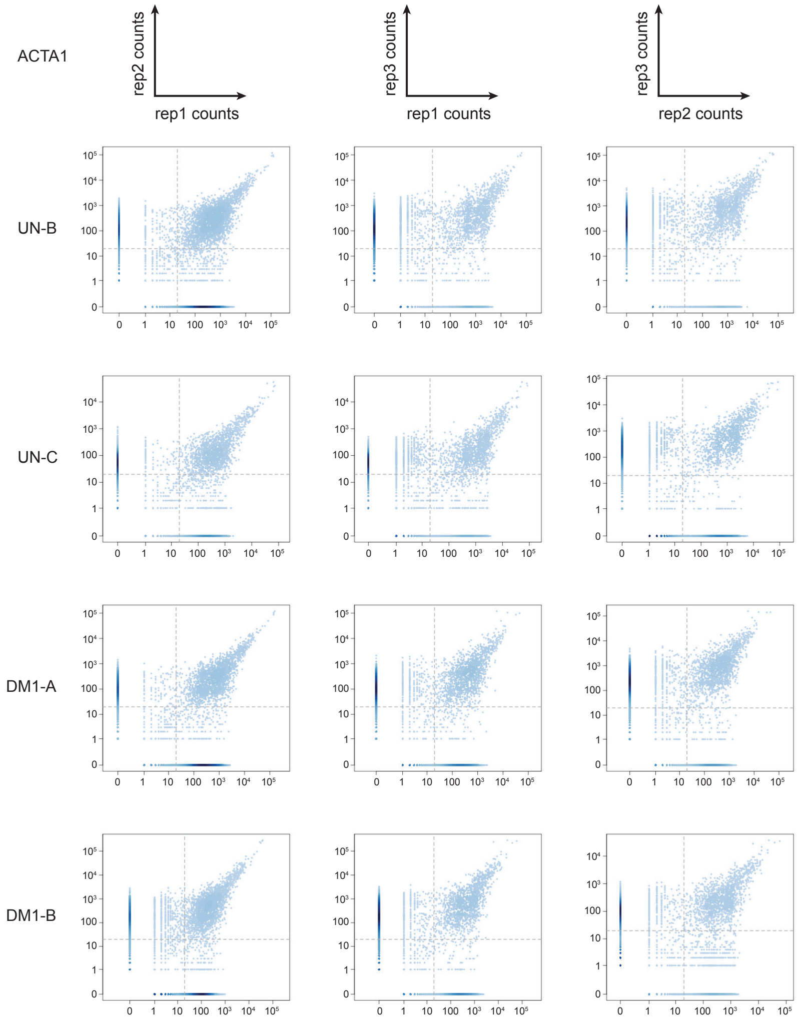
ACTA1
rep2 counts
rep1 counts
rep3 counts
rep1 counts
rep3 counts
rep2 counts
UN-B
UN-C
DM1-A
DM1-B

J

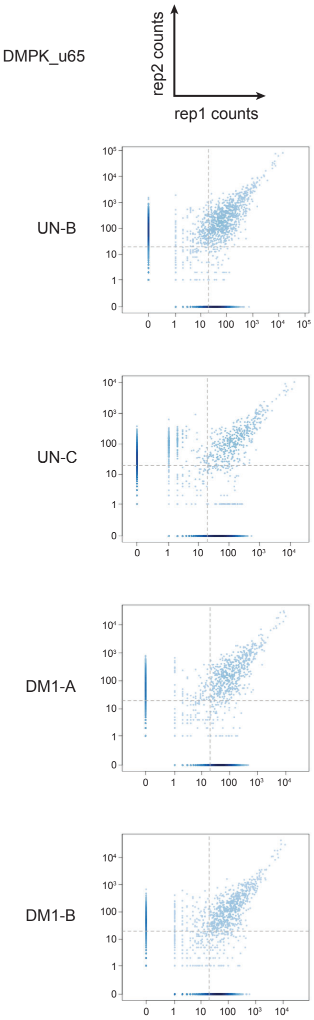
DMPK_u65
rep2 counts
rep1 counts
UN-B
UN-C
DM1-A
DM1-B

K

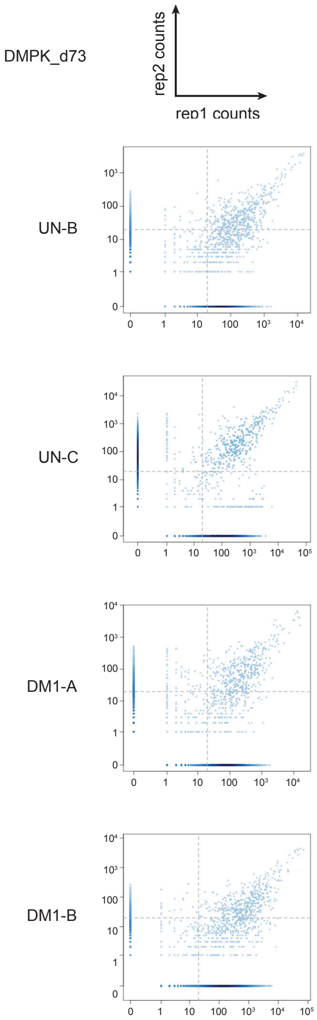
DMPK_d73
rep2 counts
rep1 counts
UN-B
UN-C
DM1-A
DM1-B

L

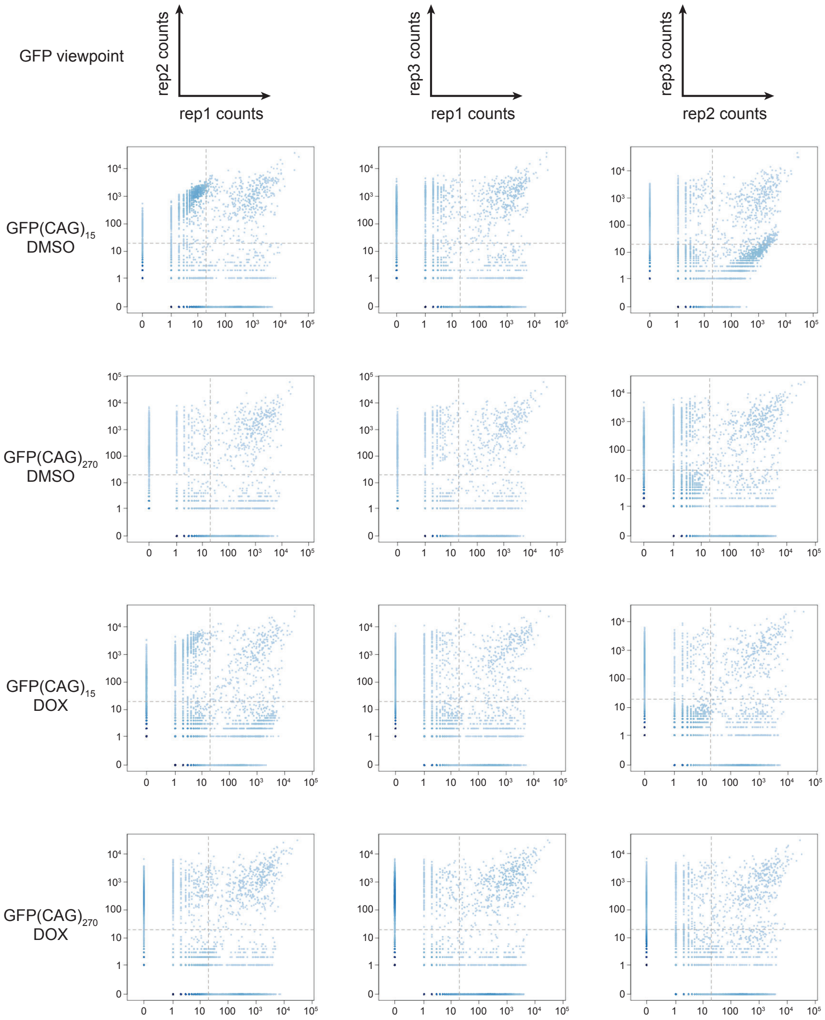
GFP viewpoint
rep2 counts
rep1 counts
rep3 counts
rep1 counts
rep3 counts
rep2 counts
GFP(CAG)15 DMSO
GFP(CAG)270 DMSO
GFP(CAG)15 DOX
GFP(CAG)270 DOX

Fig. S1. Correlation between 4C library replicates of the *FMR1*, *HTT*, *DMPK*, *ACTA1*, and GFP viewpoints.

Scatter plots comparing the mapped read counts per 4C fragment between replicates from UN-A and FXS cells **(A-C)**; from UN-B, UN-C, HD-A, HD-B, and HD-C cells **(D-F)**; from UN-B, UN-C, DM1-A, and DM1-B cells **(G-K)**; and from GFP$(CAG)_{15}$ and GFP$(CAG)_{270}$ **(L)** separated by viewpoint: **(A)** FMR1_u1 viewpoint (1 kb upstream of the CGG repeats of *FMR1*), **(B)** FMR1_u195 viewpoint (195 kb upstream of the CGG repeats of *FMR1*), **(C)** *ACTA1* viewpoint, **(D)** HTT_d1 viewpoint (1 kb downstream of the CAG repeats), **(E)** HTT_d85 viewpoint (85 kb downstream of the CAG repeats of *HTT*), **(F)** *ACTA1* viewpoint, **(G)** DMPK_d11 viewpoint (11 kb downstream of the CTG repeats of *DMPK*), **(H)** DMPK_u16 viewpoint (16 kb upstream of the CTG repeats of *DMPK*), **(I)** *ACTA1* viewpoint, **(J)** DMPK_u65 viewpoint (65 kb upstream of the CTG repeats of *DMPK*), **(K)** DMPK_d73 viewpoint (73 kb downstream of the CTG repeats of *DMPK*), and **(L)** GFP viewpoint in GFP$(CAG)_{15}$ and GFP$(CAG)_{270}$ cells with transcription off (DMSO) or on (DOX).

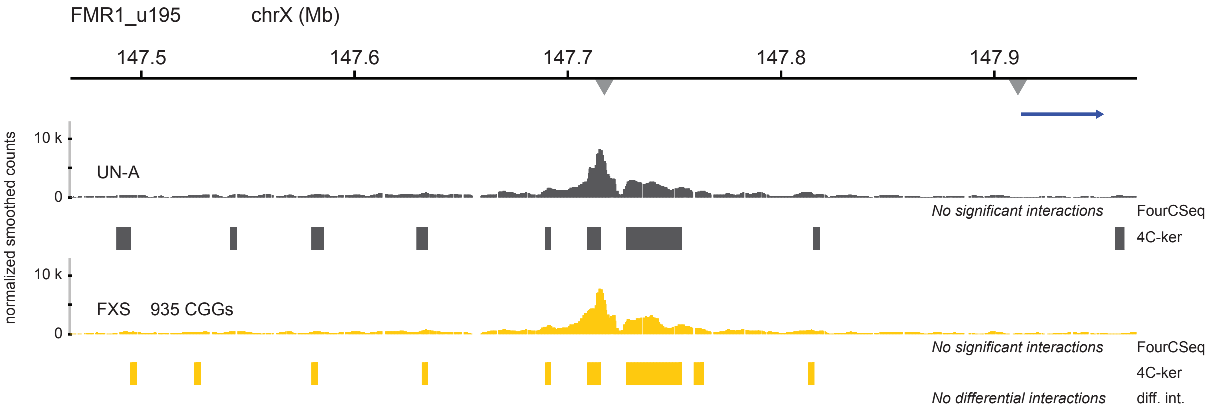

Fig. S2. Chromatin interactions of the FMR1_u195 viewpoint in unaffected and FXS patient cells. 4C-seq chromatin interaction profiles from the FMR1_u195 viewpoint (195 kb upstream of the CGG repeats of *FMR1*) in one unaffected (UN-A) and one FXS LCLs (FXS). The top blue bar represents the *FMR1* gene. The triangles at the top represent the location of the two *FMR1* viewpoints. High-interacting regions were called using 4C-ker and significant interactions were called using FourCSeq. Regions of differential interactions are marked with black bars below each 4C-seq track and labeled as “diff. int.”.

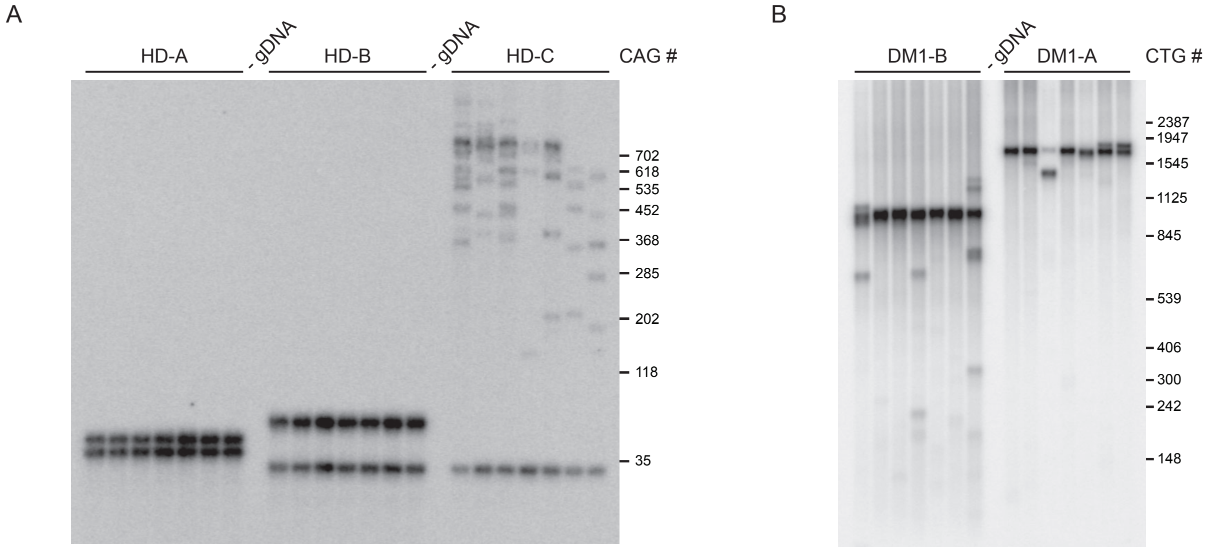

Fig. S3. Repeat sizes of HD and DM1 patient cell lines.

(A) Small-pool PCR of DNA isolated from HD-A, HD-B, and HD-C cells (shown: 1 ng gDNA per PCR). Lanes labeled "-gDNA" are control PCRs with no gDNA. **(B)** Small-pool PCR of DNA isolated from DM1-A and DM1-B cells (shown: 1 ng gDNA per PCR). Lanes labeled "-gDNA" are control PCRs with no gDNA.

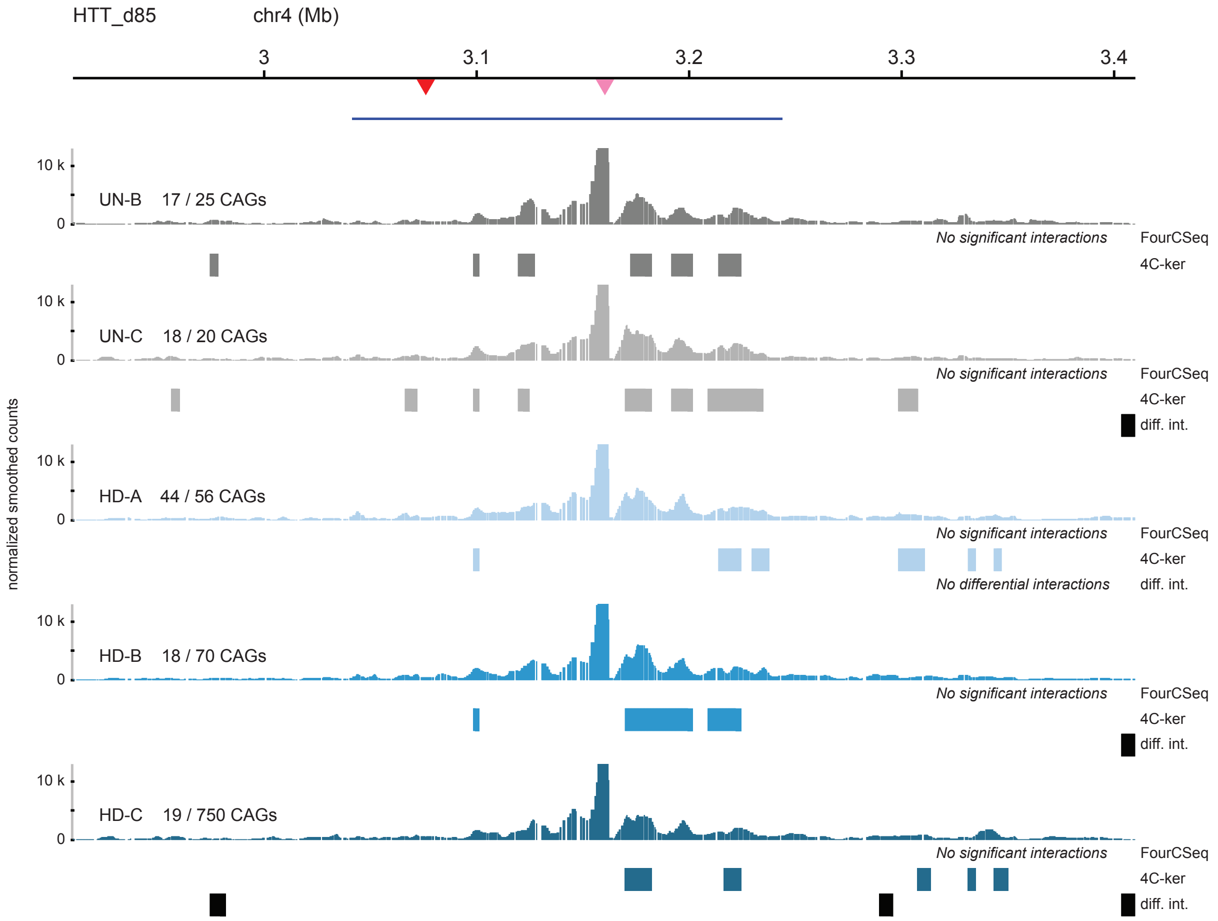

Fig. S4. Chromatin interactions of the HTT_d85 viewpoint in unaffected and HD patient cells. 4C-seq chromatin interaction profiles from the HTT_d85 viewpoint (85 kb downstream of the CAG repeats of *HTT* – central pink triangle) in two unaffected (UN-B and UN-C) and three HD LCLs (HD-A, HD-B, and HD-C). The top blue bar represents the *HTT* gene. The triangles at the top represent the location of the two *HTT* viewpoints. High-interacting regions were called using 4C-ker and significant interactions were called using FourCSeq. Regions of differential interactions compared to UN-B are marked with black bars below each 4C-seq track and labeled as “diff. int.”.

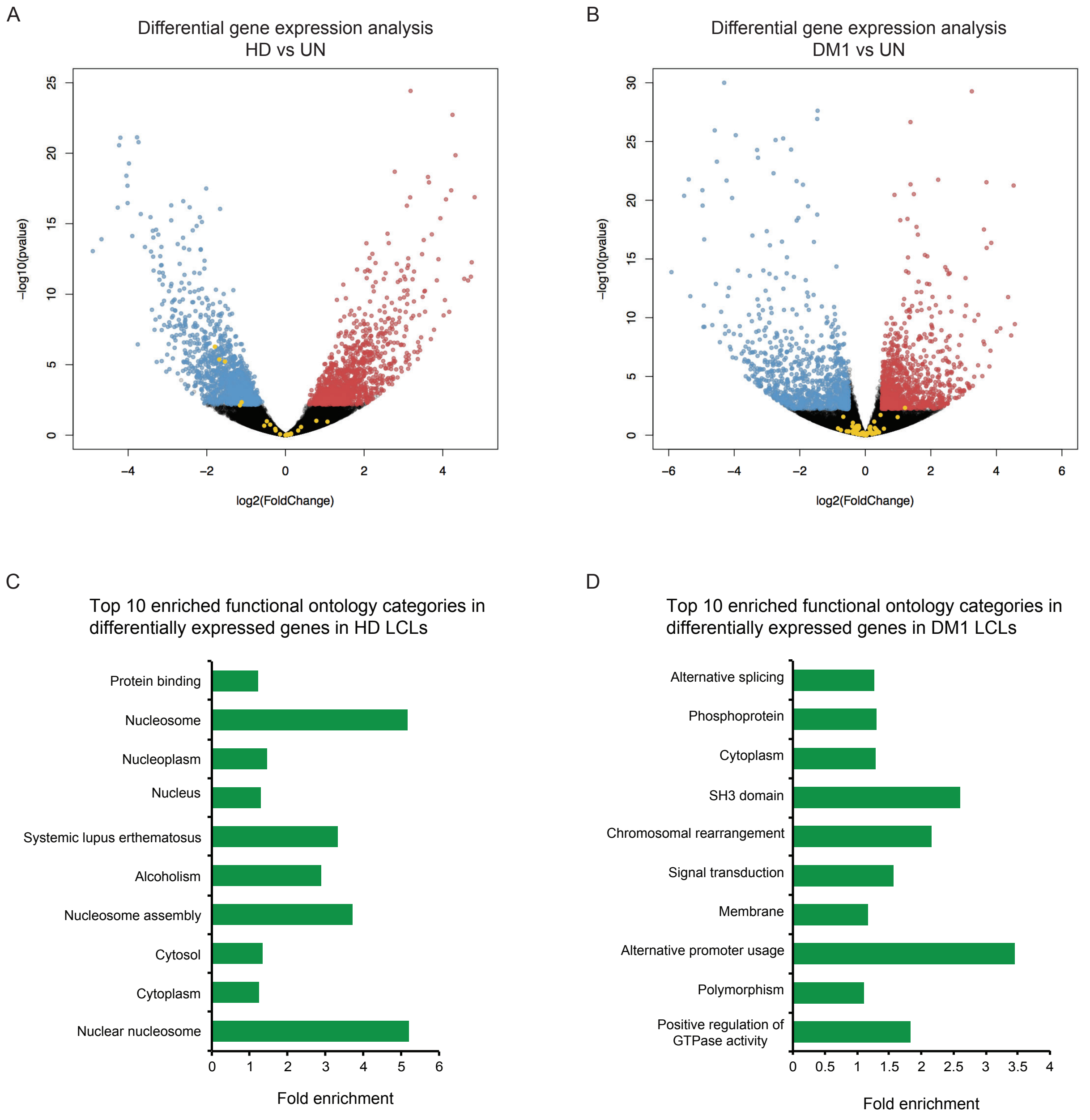

Fig. S5. Analysis of RNA-seq data from unaffected, HD, and DM1 patient cells.
(A and B) Volcano plot showing the global transcriptional differences between the **(A)** HD and **(B)** DM1 LCLs compared to the unaffected LCLs. Significantly up-regulated (red) and down-regulated (blue) genes are defined as those with log2 fold-change greater than 0.5 and an adjusted p-value less than 0.05. Genes within high-interacting regions (identified with 4C-ker) near the *HTT* and *DMPK* genes are colored in yellow. In the HD LCLs there are 1,183 significantly up-regulated genes and 1,307 significantly down-regulated genes. In the DM1 LCLs there are 1,015 significantly up-regulated genes and 949 significantly down-regulated genes. **(C and D)** Enrichment of the top 10 functional ontology terms associated with significantly differentially expressed genes in **(C)** HD and **(D)** DM1 LCLs.

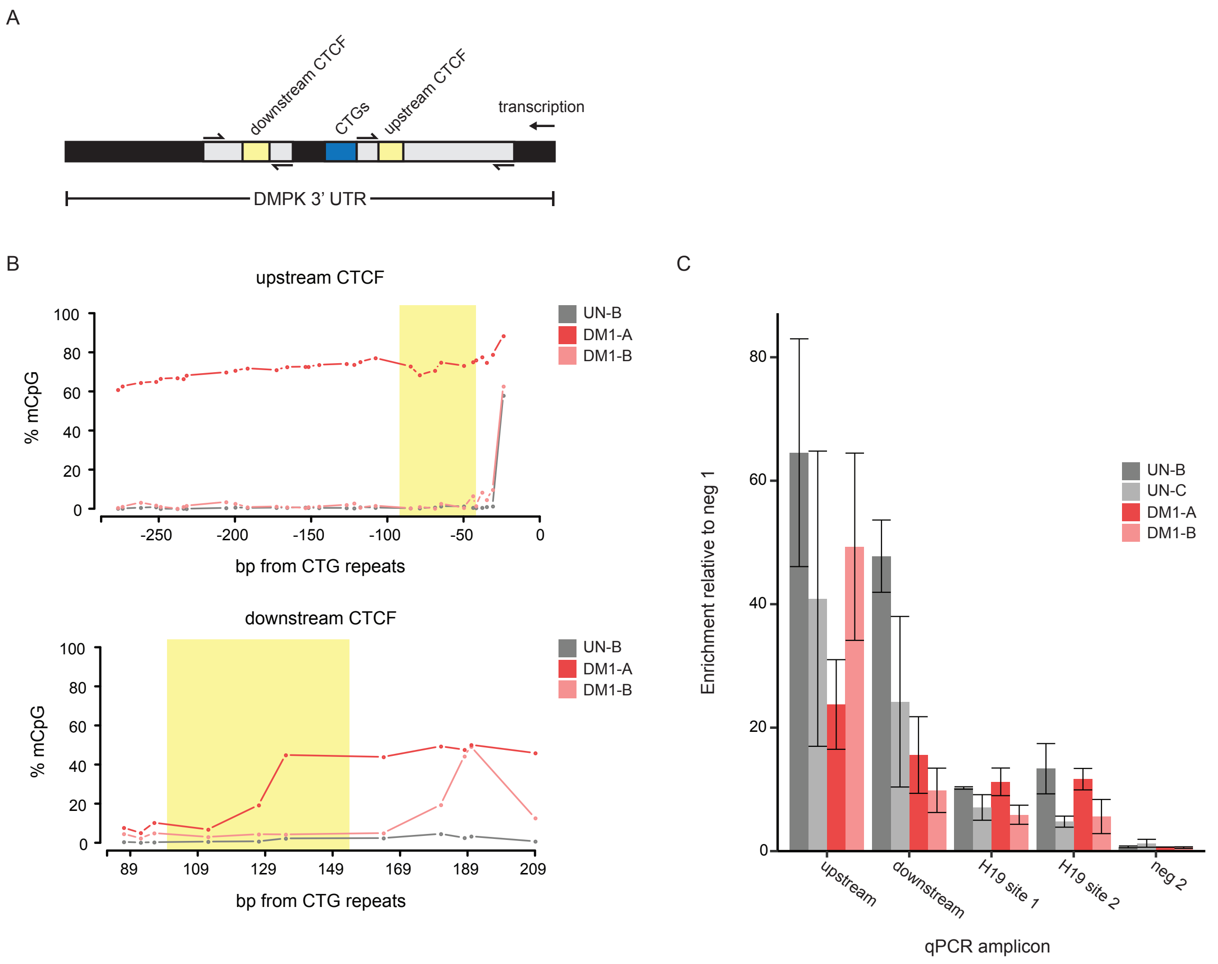

Fig. S6. Heterochromatinization of expanded *DMPK* alleles in DM1 patient cells.

(A) Schematic of the 3' UTR of *DMPK*. The PCR amplicons used for bisulfite sequencing and CTCF ChIP-qPCR are indicated in grey. The CTCF binding sites are represented by the yellow boxes. **(B)** CpG methylation levels of two CTCF binding sites directly flanking the CTG repeats of *DMPK* using bisulfite-sequencing (top: upstream site; bottom: downstream site). The specific CTCF binding sites are demarcated with yellow bars. **(C)** Chromatin immunoprecipitation of CTCF followed by qPCR at the cognate binding sites found in A and B. This was done in the UN-B, UN-C, DM1-A, and DM1-B LCLs (N=3 for all). CTCF was expected to be bound to the H19 gene, but not at two intergenic sites (neg 1 and neg 2). We normalized the enrichment at each site to neg 1. We found no significant differences when we normalized to the neg 2 site. The error bars represent the standard error of the mean.

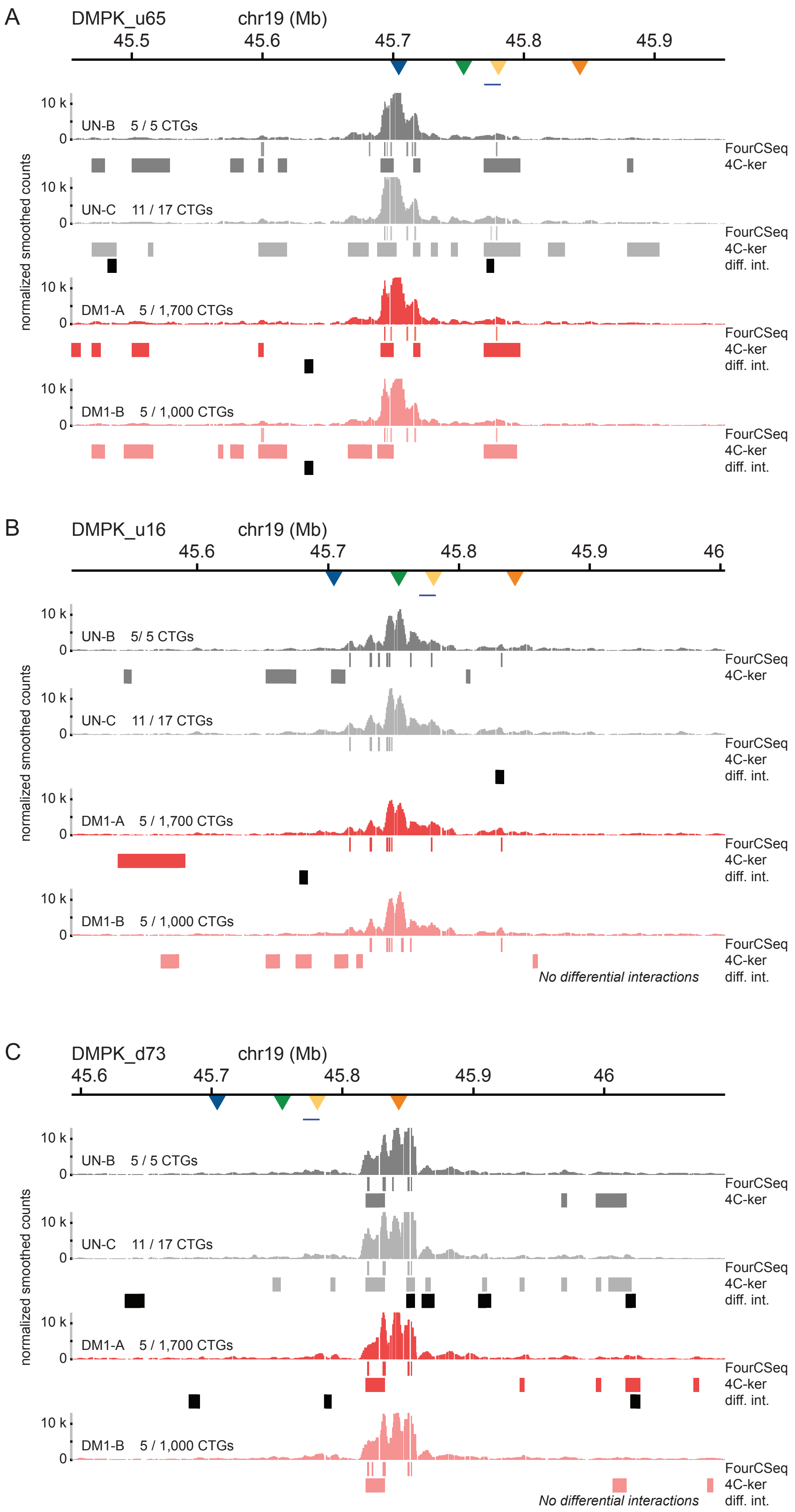
A
DMPK_u65
chr19 (Mb)
45.5
45.6
45.7
45.8
45.9
normalized smoothed counts
10 k
0
UN-B 5 / 5 CTGs
FourCSeq
4C-ker
UN-C 11 / 17 CTGs
diff. int.
DM1-A 5 / 1,700 CTGs
DM1-B 5 / 1,000 CTGs
B
DMPK_u16
chr19 (Mb)
45.6
45.7
45.8
45.9
46
UN-B 5/ 5 CTGs
UN-C 11 / 17 CTGs
DM1-A 5 / 1,700 CTGs
DM1-B 5 / 1,000 CTGs
No differential interactions
C
DMPK_d73
chr19 (Mb)
45.6
45.7
45.8
45.9
46
UN-B 5 / 5 CTGs
UN-C 11 / 17 CTGs
DM1-A 5 / 1,700 CTGs
DM1-B 5 / 1,000 CTGs
No differential interactions

Fig. S7. Chromatin interactions at DMPK_u65, DMPK_u16, and DMPK_d73 viewpoints in unaffected and DM1 patient cells.

(A) 4C-seq chromatin interaction profiles from the DMPK_u65 viewpoint (65 kb upstream of the CTG repeats of *DMPK*) in two unaffected (UN-B and UN-C) and two DM1 LCLs (DM1-A and DM1-B). **(B)** 4C-seq chromatin interaction profiles from the DMPK_u16 viewpoint (16 kb upstream of the CTG repeats of *DMPK*) in two unaffected (UN-B and UN-C) and two DM1 LCLs (DM1-A and DM1-B). **(C)** 4C-seq chromatin interaction profiles from the DMPK_d73 viewpoint (73 kb downstream of the CTG repeats of *DMPK*). For all panels, the top blue bar represents the *DMPK* gene. The triangles at the top represent the location of the four *DMPK* viewpoints. High-interacting regions were called using 4C-ker and significant interactions were called using FourCSeq. Regions of differential interactions compared to UN-B are marked with black bars below each 4C-seq track and labeled as “diff. int.”.

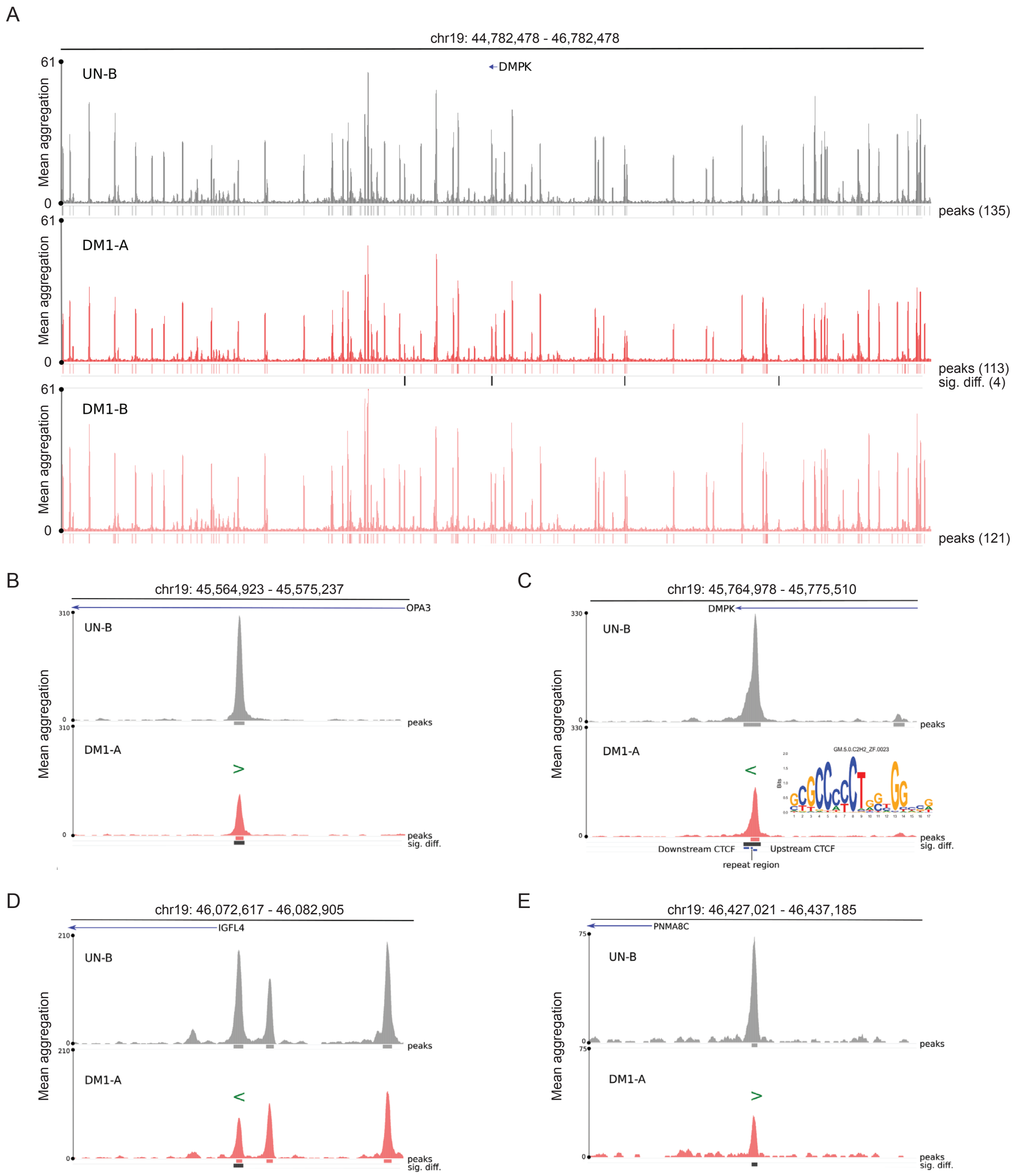

Fig. S8. CTCF occupancy in unaffected and DM1 patient cells.

(A) ChIP-seq profiles of CTCF occupancy in UN-B, DM1-A, and DM1-B cells visualized in a 2 Mb region centered around *DMPK*. The number of reproducible peaks between replicates of the same cell line (n = 3) are plotted below each ChIP-seq track. Compared to UN-B cells, four significant differentially enriched CTCF peaks (FDR < 5%) were identified in DM1-A and plotted as black bars below the CTCF peaks track. No differentially enriched CTCF peaks were identified in DM1-B cells in this genomic region. **(B-E)** ChIP-seq profiles of the significant differentially enriched CTCF peaks from DM1-A compared to UN-B cells. The inset in **(C)** represents the most enriched motif found in the reproducible peaks in DM1-A cells. The orientation of the CTCF motif is represented by the green “<” or “>” signs.

Table S1. Characteristics of cell lines used in this study.

Cell line	Code	Disease status [1]	Sex [1]	Age at sampling (years) [1]	Repeat locus	# of repeats in wt allele [2]	Sizing method	# of repeats in expanded allele [2]	Sizing method
GM09236	UN-A	unaffected	Male	4	*FMR1*	NA	Southern blot	NA	Southern blot
GM09237	FXS	FXS	Male	5	*FMR1*	5 - 45	-	935	-
GM04604	UN-B	unaffected	Male	NA	*DMPK*	5	Sanger sequencing	5	Sanger sequencing
					HTT	17	Sanger sequencing	25	Sanger sequencing
GM02180	UN-C	unaffected	Female	51	*DMPK*	11	Sanger sequencing	17	Sanger sequencing
					HTT	18	Sanger sequencing	20	Sanger sequencing
GM02164	HD-A	HD	Male	58	*HTT*	44	sp-PCR	56	sp-PCR
GM03620	HD-B	HD	Female	29	*HTT*	18	Sanger sequencing	70	sp-PCR
GM14044	HD-C	HD	Male	16	*HTT*	19	Sanger sequencing	750	sp-PCR
GM06077	DM1-A	DM1	Female	4	*DMPK*	5	Sanger sequencing	1700	sp-PCR
GM04648	DM1-B	DM1	Male	23	*DMPK*	5	Sanger sequencing	1000	sp-PCR

[1] Information obtained from the Coriell Institute Human Genetic Cell Repository.

[2] For sp-PCR, the number of repeats is an estimation of the modal number of repeats.

NA: not available

sp-PCR: small pool-PCR

Table S2. 4C-seq library statistics.

Sample	# of reads	# of mapped reads	% of total	# of reads mapped to cis chr	% of cis chr reads from total	# of reads mapped to valid 4C frags in cis chr	% of reads in 4C frags from total cis chr reads
ACTA1_GM02164_rep1	4'982'938	3'226'684	64.8	2'262'970	70.1	1'598'786	70.6
ACTA1_GM02164_rep2	14'175'182	9'482'669	66.9	6'432'203	67.8	4'689'799	72.9
ACTA1_GM02164_rep3	8'985'756	6'149'600	68.4	4'314'765	70.2	3'032'024	70.3
ACTA1_GM02180_rep1	4'445'588	2'894'160	65.1	2'047'454	70.7	1'448'565	70.7
ACTA1_GM02180_rep2	9'724'309	6'455'364	66.4	4'490'505	69.6	3'238'325	72.1
ACTA1_GM02180_rep3	14'471'204	9'623'984	66.5	6'832'538	71	5'111'636	74.8
ACTA1_GM03620_rep1	3'392'689	2'252'717	66.4	1'594'521	70.8	1'125'432	70.6
ACTA1_GM03620_rep2	10'153'899	6'911'566	68.1	4'682'012	67.7	3'412'228	72.9
ACTA1_GM03620_rep3	10'314'868	6'898'449	66.9	4'788'393	69.4	3'452'374	72.1
ACTA1_GM04604_rep1	17'462'688	9'831'359	56.3	5'908'533	60.1	4'186'550	70.9
ACTA1_GM04604_rep2	20'016'202	13'201'152	66	9'429'955	71.4	6'749'626	71.6
ACTA1_GM04604_rep3	14'370'212	9'150'078	63.7	6'493'921	71	4'854'805	74.8
ACTA1_GM04648_rep1	28'697'528	18'150'768	63.2	9'891'236	54.5	7'190'802	72.7
ACTA1_GM04648_rep2	6'247'015	4'135'124	66.2	2'850'483	68.9	2'025'028	71
ACTA1_GM04648_rep3	7'615'653	5'226'254	68.6	3'625'835	69.4	2'493'472	68.8
ACTA1_GM06077_rep1	11'677'329	7'881'675	67.5	4'502'685	57.1	3'148'013	69.9
ACTA1_GM06077_rep2	20'390'273	13'386'906	65.7	9'414'304	70.3	6'751'808	71.7
ACTA1_GM06077_rep3	6'528'425	4'468'502	68.4	3'101'465	69.4	2'186'147	70.5

ACTA1_GM09236_rep1	8'074'443	5'948'070	73.7	4'124'118	69.3	2'715'415	65.8
ACTA1_GM09236_rep2	8'888'269	6'536'752	73.5	4'465'566	68.3	3'067'787	68.7
ACTA1_GM09236_rep3	8'115'137	5'782'812	71.3	3'904'453	67.5	2'648'397	67.8
ACTA1_GM09237_rep1	8'203'008	5'994'960	73.1	4'214'919	70.3	2'743'902	65.1
ACTA1_GM09237_rep2	8'256'813	5'889'920	71.3	4'180'631	71	2'559'635	61.2
ACTA1_GM09237_rep3	7'692'644	5'546'115	72.1	3'854'904	69.5	2'386'295	61.9
ACTA1_GM14044_rep1	3'864'340	2'522'916	65.3	1'719'045	68.1	1’218'110	70.9
ACTA1_GM14044_rep2	13'566'864	8'987'115	66.2	6'029'854	67.1	4'498'420	74.6
ACTA1_GM14044_rep3	10'730'254	7'441'130	69.3	5'128'011	68.9	3'821'275	74.5
DMPK_d11_GM02180_rep1	4'296'953	866'253	20.2	442'187	51	330'779	74.8
DMPK_d11_GM02180_rep2	3'308'800	894'717	27	412'986	46.2	308'579	74.7
DMPK_d11_GM02180_rep3	7'282'955	4'517'085	62	2'175'116	48.2	1'518'060	69.8
DMPK_d11_GM04604_rep1	23'059'825	10'787'846	46.8	2'388'136	22.1	1'782'296	74.6
DMPK_d11_GM04604_rep2	8'042'866	717'825	8.9	355'974	49.6	251'617	70.7
DMPK_d11_GM04604_rep3	6'716'905	4'244'006	63.2	1'416'961	33.4	988'977	69.8
DMPK_d11_GM04648_rep1	8'560'029	4'972'802	58.1	1'413'824	28.4	979'700	69.3
DMPK_d11_GM04648_rep2	12'454'988	1'910'083	15.3	928'582	48.6	678'608	73.1
DMPK_d11_GM04648_rep3	8'259'430	3'846'132	46.6	1'529'463	39.8	1'063'240	69.5
DMPK_d11_GM06077_rep1	15'395'452	7'880'443	51.2	2'460'619	31.2	1'843'809	74.9
DMPK_d11_GM06077_rep2	774'586	109'054	14.1	56'680	52	41'488	73.2

DMPK_d11_GM06077_rep3	6'524'825	2'605'272	39.9	1'097'272	42.1	773'163	70.5
DMPK_d73_GM02180_rep1	4'278'924	2'351'635	55	781'457	33.2	512'019	65.5
DMPK_d73_GM02180_rep2	6'211'002	4'265'102	68.7	1'463'307	34.3	918'581	62.8
DMPK_d73_GM04604_rep1	5'976'375	5'457'650	91.3	325'342	6	171'443	52.7
DMPK_d73_GM04604_rep2	4'710'733	2'209'035	46.9	816'530	37	507'446	62.1
DMPK_d73_GM04648_rep1	8'317'618	7'753'683	93.2	686'800	8.9	210'742	30.7
DMPK_d73_GM04648_rep2	13'833'430	8'816'046	63.7	3'351'872	38	2'184'265	65.2
DMPK_d73_GM06077_rep1	25'744'531	24'078'279	93.5	2'287'837	9.5	334'267	14.6
DMPK_d73_GM06077_rep2	5'923'808	2'670'770	45.1	871'927	32.6	565'589	64.9
DMPK_u16_GM02180_rep1	4'967'776	3'272'865	65.9	1'156'773	35.3	807'405	69.8
DMPK_u16_GM02180_rep2	9'791'581	6'074'072	62	2'791'710	46	1'918'545	68.7
DMPK_u16_GM02180_rep3	3'499'397	2'404'710	68.7	743'880	30.9	494'040	66.4
DMPK_u16_GM04604_rep1	16'969'035	13'541'809	79.8	2'368'499	17.5	1'502'637	63.4
DMPK_u16_GM04604_rep2	8'471'505	1'910'469	22.6	611'665	32	391'201	64
DMPK_u16_GM04604_rep3	5'023'436	4'174'649	83.1	325'026	7.8	214'817	66.1
DMPK_u16_GM04648_rep1	14'372'890	11'367'448	79.1	1'616'380	14.2	1'080'070	66.8
DMPK_u16_GM04648_rep2	5'456'784	1'941'577	35.6	874'656	45	560'803	64.1
DMPK_u16_GM04648_rep3	8'354'943	5'260'739	63	2'365'753	45	1'549'651	65.5
DMPK_u16_GM06077_rep1	44'300'878	31'633'701	71.4	4'230'357	13.4	3'136'434	74.1

DMPK_u16_GM06077_rep2	7'516'556	2'615'231	34.8	791'991	30.3	512'797	64.7
DMPK_u16_GM06077_rep3	3'344'272	2'426'791	72.6	693'947	28.6	436'064	62.8
DMPK_u65_GM02180_rep1	1'183'618	725'020	61.3	402'692	55.5	342'602	85.1
DMPK_u65_GM02180_rep2	1'461'840	888'805	60.8	466'817	52.5	406'381	87.1
DMPK_u65_GM04604_rep1	11'192'157	8'116'207	72.5	2'296'664	28.3	1'805'386	78.6
DMPK_u65_GM04604_rep2	1'688'853	1'035'697	61.3	606'020	58.5	513'147	84.7
DMPK_u65_GM04648_rep1	8'646'819	5'368'775	62.1	1'045'112	19.5	942'967	90.2
DMPK_u65_GM04648_rep2	2'167'574	1'221'577	56.4	668'154	54.7	588'914	88.1
DMPK_u65_GM06077_rep1	8'498'158	5'535'188	65.1	1'053'071	19	908'403	86.3
DMPK_u65_GM06077_rep2	1'586'296	966'005	60.9	574'031	59.4	515'578	89.8
FMR1_u1_GM09236_rep1	8'348'904	6'663'081	79.8	4'745'063	71.2	3'213'100	67.7
FMR1_u1_GM09236_rep2	9'971'410	8'071'773	80.9	5'499'110	68.1	3'732'174	67.9
FMR1_u1_GM09236_rep3	7'640'685	6'321'818	82.7	4'460'210	70.6	2'982'866	66.9
FMR1_u1_GM09237_rep1	7'111'884	5'790'600	81.4	4'289'120	74.1	2'717'005	63.3
FMR1_u1_GM09237_rep2	9'404'577	7'747'635	82.4	5'810'983	75	3'931'135	67.7
FMR1_u1_GM09237_rep3	8'724'375	7'372'925	84.5	5'458'328	74	3'622'857	66.4
FMR1_u195_GM09236_rep1	6'435'220	5'040'291	78.3	3'762'717	74.7	2'584'632	68.7
FMR1_u195_GM09236_rep2	7'539'577	6'039'207	80.1	4'279'877	70.9	2'968'395	69.4

FMR1_u195_GM09236_rep3	6'160'874	5'006'543	81.3	3'536'109	70.6	2'479'535	70.1
FMR1_u195_GM09237_rep1	6'558'723	5'366'966	81.8	3'873'507	72.2	2'627'038	67.8
FMR1_u195_GM09237_rep2	8'285'584	6'011'108	72.5	4'371'687	72.7	2'997'459	68.6
FMR1_u195_GM09237_rep3	9'774'942	7'913'166	81	5'558'279	70.2	3'884'446	69.9
GFP_CAG_15_DMSO_rep1	7'048'343	2'833'417	40.2	1'700'523	60	1'278'717	75.2
GFP_CAG_15_DMSO_rep2	7'487'213	2'887'212	38.6	1'638'799	56.8	1'284'684	78.4
GFP_CAG_15_DMSO_rep3	6'936'377	2'769'339	39.9	1'464'325	52.9	1'172'916	80.1
GFP_CAG_15_DOX_rep1	11'304'727	4'562'245	40.4	2'182'322	47.8	1'705'839	78.2
GFP_CAG_15_DOX_rep2	8'452'926	3'124'374	37	1'540'855	49.3	1'198'057	77.8
GFP_CAG_15_DOX_rep3	7'631'549	2'833'020	37.1	1'466'250	51.8	1'157'955	79
GFP_CAG_270_DMSO_rep1	8'320'728	3'099'916	37.3	1'907'976	61.5	1'512'580	79.3
GFP_CAG_270_DMSO_rep2	6'840'505	2'694'631	39.4	1'575'886	58.5	1'229'060	78
GFP_CAG_270_DMSO_rep3	7'338'988	2'673'561	36.4	1'569'149	58.7	1'225'397	78.1
GFP_CAG_270_DOX_rep1	9'301'866	3'679'030	39.6	2'078'639	56.5	1'620'884	78
GFP_CAG_270_DOX_rep2	8'353'878	3'289'609	39.4	1'735'082	52.7	1'333'717	76.9
GFP_CAG_270_DOX_rep3	7'577'290	3'045'799	40.2	1'588'724	52.2	1'267'573	79.8
HTT_d1_GM02164_rep1	1'547'564	1'006'519	65	550'667	54.7	378'050	68.7
HTT_d1_GM02164_rep2	6'426'843	4'114'843	64	2'049'276	49.8	1'475'404	72
HTT_d1_GM02164_rep3	4'561'062	2'649'147	58.1	1'529'524	57.7	1'141'625	74.6

HTT_d1_GM02180_rep1	2'641'610	1'678'631	63.5	975'311	58.1	687'885	70.5
HTT_d1_GM02180_rep2	4'261'764	2'775'669	65.1	1'331'970	48	972'583	73
HTT_d1_GM02180_rep3	6'713'195	3'878'698	57.8	2'112'225	54.5	1'644'256	77.8
HTT_d1_GM03620_rep1	2'799'272	1'795'212	64.1	1'031'035	57.4	704'440	68.3
HTT_d1_GM03620_rep2	5'475'190	3'487'162	63.7	1'558'114	44.7	1'137'993	73
HTT_d1_GM03620_rep3	7'098'155	3'663'907	51.6	2'006'314	54.8	1'576'201	78.6
HTT_d1_GM04604_rep1	4'212'566	2'457'650	58.3	1'404'970	57.2	1'103'125	78.5
HTT_d1_GM04604_rep2	10'749'708	1'832'850	17.1	888'698	48.5	639'666	72
HTT_d1_GM04604_rep3	5'921'707	3'530'799	59.6	2'010'544	56.9	1'556'448	77.4
HTT_d1_GM14044_rep1	2'595'315	1'668'384	64.3	945'665	56.7	659'221	69.7
HTT_d1_GM14044_rep2	6'212'554	3'953'292	63.6	1'666'102	42.1	1'189'773	71.4
HTT_d1_GM14044_rep3	5'199'603	2'755'357	53	1'459'570	53	1'128'283	77.3
HTT_d85_GM02164_rep1	2'065'583	773'630	37.5	440'336	56.9	279'660	63.5
HTT_d85_GM02164_rep2	4'271'456	2'674'960	62.6	1'320'008	49.3	846'436	64.1
HTT_d85_GM02164_rep3	3'365'643	1'762'199	52.4	1'048'467	59.5	674'809	64.4
HTT_d85_GM02180_rep1	2'884'811	1'333'135	46.2	773'017	58	467'647	60.5
HTT_d85_GM02180_rep2	4'805'661	3'283'731	68.3	1'132'457	34.5	719'942	63.6
HTT_d85_GM02180_rep3	3'937'446	2'278'255	57.9	1'335'882	58.6	909'086	68.1
HTT_d85_GM03620_rep1	3'134'531	654'436	20.9	397'137	60.7	245'017	61.7
HTT_d85_GM03620_rep2	3'232'273	2'024'049	62.6	978'716	48.4	577'818	59

HTT_d85_GM03620_rep3	4'074'032	2'290'232	56.2	1'356'058	59.2	898'455	66.3
HTT_d85_GM04604_rep1	3'626'944	2'088'253	57.6	1'300'447	62.3	850'403	65.4
HTT_d85_GM04604_rep2	4'387'441	2'691'863	61.4	1'062'983	39.5	637'080	59.9
HTT_d85_GM04604_rep3	4'022'177	2'243'116	55.8	1'343'423	59.9	882'695	65.7
HTT_d85_GM14044_rep1	2'057'402	798'621	38.8	453'565	56.8	276'126	60.9
HTT_d85_GM14044_rep2	5'576'017	2'543'284	45.6	956'326	37.6	583'814	61
HTT_d85_GM14044_rep3	3'538'845	2'077'028	58.7	1'191'091	57.3	772'206	64.8

Table S5. PCR primers used in this study.

Name	Application	Viewpoint / amplicon	Primer type	5’ - 3’ primer sequence	Reference
oVIN-1333	*HTT* CAG repeats	*HTT* CAG repeats	Forward	CCGCTCAGGTTCTGCTTTTA	This study
oVIN-1334	*HTT* CAG repeats	*HTT* CAG repeats	Reverse	CAGGCTGCAGGGTTACCG	This study
oVIN-1252	*DMPK* CTG repeats	*DMPK* CTG repeats	Forward	CACTTTGCGAACCAACGATA	This study
oVIN-1251	*DMPK* CTG repeats	*DMPK* CTG repeats	Reverse	GAGCGTGGGTCTCCGCCCAG	This study
oVIN-2103	Bisulfite seq PCR 1	upstream CTCF site	Forward	TGTYGTYGTTTTGGGTTGTATTG	*(32)*
oVIN-2110	Bisulfite seq PCR 1	upstream CTCF site	Reverse	CAACATTCCYGACTACAAAAACCCTT	*(32)*
oVIN-2107	Bisulfite seq PCR 1	downstream CTCF site	Forward	TTYGGTTAGGTTGAGGTTT	*(32)*
oVIN-2108	Bisulfite seq PCR 1	downstream CTCF site	Reverse	TTAACAAAAACAAATTTCCC	*(32)*
oVIN-2111	Bisulfite seq PCR 2	upstream CTCF site	Forward	TCGTCGGCAGCGTCAGATGTGTATAAGAGACAGGTT GTATTGGGTTGGTGGTTTA	*(32)*
oVIN-2112	Bisulfite seq PCR 2	upstream CTCF site	Reverse	GTCTCGTGGGCTCGGAGATGTGTATAAGAGACAGCT ACAAAAACCCTTYGAACCC	*(32)*

oVIN-2113	Bisulfite seq PCR 2	downstream CTCF site	Forward	TCGTCGGCAGCGTCAGATGTGTATAAGAGACAGTAAATTGTAGGTTTGGGAAG	*(32)*
oVIN-2114	Bisulfite seq PCR 2	downstream CTCF site	Reverse	GTCTCGTGGGCTCGGAGATGTGTATAAGAGACAGTTAACAAAAACAAATTTCCC	*(32)*
oVIN-2443	CTCF ChIP qPCR	upstream CTCF site	Forward	AAGGACCCTTCGAGCCCC	This study
oVIN-2444	CTCF ChIP qPCR	upstream CTCF site	Reverse	AGTTCACAACCGCTCCGAG	This study
oVIN-2445	CTCF ChIP qPCR	downstream CTCF site	Forward	GCAAAAGCAAATTTCCCGAGT	This study
oVIN-2446	CTCF ChIP qPCR	downstream CTCF site	Reverse	AAACTGCAGGCCTGGGAA	This study
oVIN-2447	CTCF ChIP qPCR	H19 site 1	Forward	CCCATCTTGCTGACCTCAC	ChIPAb+ CTCF - (Millipore)
oVIN-2448	CTCF ChIP qPCR	H19 site 1	Reverse	AGACCTGGGACGTTTCTGTG	ChIPAb+ CTCF - (Millipore)
oVIN-2556	CTCF ChIP qPCR	H19 site 2	Forward	TGTGGATAATGCCCGACCTGAAGATCTG	*(60)*
oVIN-2557	CTCF ChIP qPCR	H19 site 2	Reverse	ACGGAATTGGTTGTAGTTGTGGAATCGGAAGT	*(60)*
oVIN-976	CTCF ChIP qPCR	negative 1	Forward	CCTTCAGAACCCTTCAGTGC	This study
oVIN-977	CTCF ChIP qPCR	negative 1	Reverse	ATCACACCTGTGGCTTCCTC	This study
oVIN-978	CTCF ChIP qPCR	negative 2	Forward	TGCTTTTCTAGGAGGTAGGTGTC	This study

oVIN-979	CTCF ChIP qPCR	negative 2	Reverse	AGCCCAGCAGAAAGGTCTTC	This study
oVIN-1180	4C-seq	ACTA1	Forward (reading)	AGAACAGGCCCTTGAGGGAT	This study
oVIN-1181	4C-seq	ACTA1	Reverse (non-reading)	GGGCCATCTCCCACAGTTTG	This study
oVIN-1146	4C-seq	DMPK_d11	Forward (reading)	CCCAAAGTTGTCCCTCCTGG	This study
oVIN-1147	4C-seq	DMPK_d11	Reverse (non-reading)	CCAGGCTGCCCAGTTTAACA	This study
oVIN-1158	4C-seq	DMPK_d73	Forward (reading)	GCTGAGAGATGGAGAAATGCAGA	This study
oVIN-1159	4C-seq	DMPK_d73	Reverse (non-reading)	GTTGTTGGCTGAGGAAGGGG	This study
oVIN-1128	4C-seq	DMPK_u16	Forward (reading)	ACAAAGAGGCCTGTGATTGAGG	This study
oVIN-1129	4C-seq	DMPK_u16	Reverse (non-reading)	CTGGGTGACCTGGGCTTCTA	This study
oVIN-1136	4C-seq	DMPK_u65	Forward (reading)	GACATTGCATGACAAGGGGGA	This study
oVIN-1137	4C-seq	DMPK_u65	Reverse (non-reading)	ACTGACACAACACAAACCACAGA	This study
oVIN-2808	4C-seq	FMR1_u1	Forward (reading)	AGGAAAGGCTCACATTTTGA	This study
oVIN-2780	4C-seq	FMR1_u1	Reverse (non-reading)	TGCCAGCATTGAACTTAGTA	This study
oVIN-2799	4C-seq	FMR1_u195	Forward (reading)	CAAATGACATCCAGGCTACT	This study
oVIN-2760	4C-seq	FMR1_u195	Reverse (non-reading)	ACCAAACTATATGCAAGGTATTATC	This study
oVIN-1380	4C-seq	GFP	Forward (reading)	ATGGTGGCGACCGGTGGATC	This study
oVIN-1381	4C-seq	GFP	Reverse (non-reading)	TCTTAGGCACCTTTGTCCTA	This study
oVIN-943	4C-seq	HTT_d1	Forward (reading)	TAGGCTTAGATGAGCAGATC	*(61)*
oVIN-944	4C-seq	HTT_d1	Reverse (non-reading)	GTCAGGCTTGCCAGAATAC	*(61)*

oVIN-1030	4C-seq	HTT_d85	Forward (reading)	TGGTTGTACATTATGAGATC	*(61)*
oVIN-1031	4C-seq	HTT_d85	Reverse (non-reading)	AAAGGGTTAAATGTCCATCA	*(61)*
oVIN-2582	TLA	GFP_d3	Forward (reading)	CACAAATCAGCGATTTCCAT	This study
oVIN-2583	TLA	GFP_d3	Reverse (non-reading)	TCAGAAGCCATAGAGCCC	This study
oVIN-2590	TLA	GFP_u4	Forward (reading)	ACACAGGAAACAGCTATGAC	This study
oVIN-2589	TLA	GFP_u4	Reverse (non-reading)	TCCACAGAATCAGGGGATAA	This study

REFERENCES AND NOTES

1. J. A. Beagan, J. E. Phillips-Cremins, On the existence and functionality of topologically associating domains. *Nat. Genet.* **52**, 8–16 (2020).

2. W. A. Bickmore, B. van Steensel, Genome architecture: Domain organization of interphase chromosomes. *Cell* **152**, 1270–1284 (2013).

3. R. Kempfer, A. Pombo, Methods for mapping 3D chromosome architecture. *Nat. Rev. Genet.* **21**, 207–226 (2020).

4. D. M. Ibrahim, S. Mundlos, Three-dimensional chromatin in disease: What holds us together and what drives us apart? *Curr. Opin. Cell Biol.* **64**, 1–9 (2020).

5. V. Dion, J. H. Wilson, Instability and chromatin structure of expanded trinucleotide repeats. *Trends Genet.* **25**, 288–297 (2009).

6. A. L. Castel, J. D. Cleary, C. E. Pearson, Repeat instability as the basis for human diseases and as a potential target for therapy. *Nat. Rev. Mol. Cell Biol.* **11** 165–170 (2010).

7. D. Kumari, K. Usdin, Chromatin remodeling in the noncoding repeat expansion diseases. *J. Biol. Chem.* **284**, 7413–7417 (2009).

8. Y. H. Fu, D. P. A. Kuhl, A. Pizzuti, M. Pieretti, J. S. Sutcliffe, S. Richards, A. J. M. H. Verkert, J. J. A. Holden, R. G. Fenwick Jr, S. T. Warren, B. A. Oostra, D. L. Nelson, C. T. Caskey, Variation of the CGG repeat at the fragile X site results in genetic instability: Resolution of the Sherman paradox. *Cell* **67**, 1047–1058 (1991).

9. A. J. M. H. Verkerk, M. Pieretti, J. S. Sutcliffe, Y.-H. Fu, D. P. A. Kuhl, A. Pizzuti, O. Reiner, S. Richards, M. F. Victoria, F. Zhang, B. E. Eussen, G.-J. B. van Ommen, L. A. J. Blonden, G. J. Riggins, J. L. Chastain, C. B. Kunst, H. Galjaard, C. Thomas Caskey, D. L. Nelson, B. A. Oostra, S. T. Warran, Identification of a gene (FMR-1) containing a CGG repeat coincident with a breakpoint cluster region exhibiting length variation in fragile X syndrome. *Cell* **65**, 905–914 (1991).

10. I. Oberlé, F. Rousseau, D. Heitz, C. Kretz, D. Devys, A. Hanauer, J. Boué, M. F. Bertheas, J. L. Mandel, Instability of a 550-base pair DNA segment and abnormal methylation in fragile X syndrome. *Science* **252**, 1097–1102 (1991).

11. V. Campuzano, L. Montermini, M. D. Moltò, L. Pianese, M. Cossée, F. Cavalcanti, E. Monros, F. Rodius, F. Duclos, A. Monticelli, F. Zara, J. Cañizares, H. Koutnikova, S. I. Bidichandani, C. Gellera, A. Brice, P. Trouillas, G. De Michele, A. Filla, R. De Frutos, F. Palau, P. I. Patel, S. Di Donato, J.-L. Mandel, S. Cocozza, M. Koenig, M. Pandolfo, Friedreich's ataxia: Autosomal recessive disease caused by an intronic GAA triplet repeat expansion. *Science* **271**, 1423–1427 (1996).

12. D. Kumari, K. Usdin, The distribution of repressive histone modifications on silenced *FMR1* alleles provides clues to the mechanism of gene silencing in fragile X syndrome. *Hum. Mol. Genet.* **19**, 4634–4642 (2010).

13. N. Gheldof, T. M. Tabuchi, J. Dekker, The active FMR1 promoter is associated with a large domain of altered chromatin conformation with embedded local histone modifications. *Proc. Natl. Acad. Sci. U.S.A.* **103**, 12463–12468 (2006).

14. R. Pietrobono, E. Tabolacci, F. Zalfa, I. Zito, A. Terracciano, U. Moscato, C. Bagni, B. Oostra, P. Chiurazzi, G. Neri, Molecular dissection of the events leading to inactivation of the FMR1 gene. *Hum. Mol. Genet.* **14**, 267–277 (2005).

15. B. Coffee, F. Zhang, S. Ceman, S. T. Warren, D. Reines, Histone modifications depict an aberrantly heterochromatinized FMR1 gene in fragile X syndrome. *Am. J. Hum. Genet.* **71**, 923–932 (2002).

16. B. Coffee, F. Zhang, S. T. Warren, D. Reines, Acetylated histones are associated with FMR1 in normal but not fragile X-syndrome cells. *Nat. Genet.* **22**, 98–101 (1999).

17. P. K. Chan, R. Torres, C. Yandim, P. P. Law, S. Khadayate, M. Mauri, C. Grosan, N. Chapman-Rothe, P. Giunti, M. Pook, R. Festenstein, Heterochromatinization induced by GAA-repeat hyperexpansion in Friedreich's ataxia can be reduced upon HDAC inhibition by vitamin B3. *Hum. Mol. Genet.* **22**, 2662–2675 (2013).

18. M. V. Evans-Galea, N. Carrodus, S. M. Rowley, L. A. Corben, G. Tai, R. Saffery, J. C. Galati, N. C. Wong, J. M. Craig, D. R. Lynch, S. R. Regner, A. F. D. Brocht, S. L. Perlman, K. O. Bushara, C. M. Gomez, G. R. Wilmot, L. Li, E. Varley, M. B. Delatycki, J. P. Sarsero, FXN methylation predicts expression and clinical outcome in Friedreich ataxia. *Ann. Neurol.* **71**, 487–497 (2012).

19. I. De Biase, Y. K. Chutake, P. M. Rindler, S. I. Bidichandani, Epigenetic silencing in Friedreich ataxia is associated with depletion of CTCF (CCCTC-binding factor) and antisense transcription. *PLOS ONE* **4**, e7914 (2009).

20. S. Al-Mahdawi, R. M. Pinto, O. Ismail, D. Varshney, S. Lymperi, C. Sandi, D. Trabzuni, M. Pook, The Friedreich ataxia GAA repeat expansion mutation induces comparable epigenetic changes in human and transgenic mouse brain and heart tissues. *Hum. Mol. Genet.* **17**, 735–746 (2008).

21. E. Greene, L. Mahishi, A. Entezam, D. Kumari, K. Usdin, Repeat-induced epigenetic changes in intron 1 of the frataxin gene and its consequences in Friedreich ataxia. *Nucleic Acids Res.* **35**, 3383–3390 (2007).

22. J. H. Sun, L. Zhou, D. J. Emerson, S. A. Phyo, K. R. Titus, W. Gong, T. G. Gilgenast, J. A. Beagan, B. L. Davidson, F. Tassone, J. E. Phillips-Cremins, Disease-associated short tandem repeats co-localize with chromatin domain boundaries. *Cell* **175**, 224–238.e15 (2018).

23. A. L. Castel, M. Nakamori, S. Tomé, D. Chitayat, G. Gourdon, C. A. Thornton, C. E. Pearson, Expanded CTG repeat demarcates a boundary for abnormal CpG methylation in myotonic dystrophy patient tissues. *Hum. Mol. Genet.* **20**, 1–15 (2011).

24. D. H. Cho, C. P. Thienes, S. E. Mahoney, E. Analau, G. N. Filippova, S. J. Tapscott, Antisense transcription and heterochromatin at the DM1 CTG repeats are constrained by CTCF. *Mol. Cell* **20**, 483–489 (2005).

25. G. N. Filippova, C. P. Thienes, B. H. Penn, D. H. Cho, Y. J. Hu, J. M. Moore, T. R. Klesert, V. V. Lobanenkov, S. J. Tapscott, CTCF-binding sites flank CTG/CAG repeats and form a methylation-sensitive insulator at the DM1 locus. *Nat. Genet.* **28**, 335–343 (2001).

26. J. R. Brouwer, A. Huguet, A. Nicole, A. Munnich, G. Gourdon, Transcriptionally repressive chromatin remodelling and CpG methylation in the presence of expanded CTG-repeats at the DM1 locus. *J. Nucleic Acids*. **2013**, 567435 (2013).

27. A. D. Otten, S. J. Tapscott, Triplet repeat expansion in myotonic dystrophy alters the adjacent chromatin structure. *Proc. Natl. Acad. Sci. U.S.A.* **92**, 5465–5469 (1995).

28. X. A. Su, V. Dion, S. M. Gasser, C. H. Freudenreich, Regulation of recombination at yeast nuclear pores controls repair and triplet repeat stability. *Genes Dev.* **29**, 1006–1017 (2015).

29. E. Splinter, E. de Wit, H. J. G. van de Werken, P. Klous, W. de Laat, Determining long-range chromatin interactions for selected genomic sites using 4C-seq technology: From fixation to computation. *Methods* **58**, 221–230 (2012).

30. F. A. Klein, T. Pakozdi, S. Anders, Y. Ghavi-Helm, E. E. M. Furlong, W. Huber, FourCSeq: Analysis of 4C sequencing data. *Bioinformatics* **31**, 3085–3091 (2015).

31. R. Raviram, P. P. Rocha, C. L. Müller, E. R. Miraldi, S. Badri, Y. Fu, E. Swanzey, C. Proudhon, V. Snetkova, R. Bonneau, J. A. Skok, 4C-ker: A method to reproducibly identify genome-wide interactions captured by 4C-seq experiments. *PLOS Comput. Biol.* **12**, e1004780 (2016).

32. L. Barbé, S. Lanni, A. López-Castel, S. Franck, C. Spits, K. Keymolen, S. Seneca, S. Tomé, I. Miron, J. Letourneau, M. Liang, S. Choufani, R. Weksberg, M. D. Wilson, Z. Sedlacek, C. Gagnon, Z. Musova, D. Chitayat, P. Shannon, J. Mathieu, K. Sermon, C. E. Pearson, CpG methylation, a parent-of-origin effect for maternal-biased transmission of congenital myotonic dystrophy. *Am. J. Hum. Genet.* **100**, 488–505 (2017).

33. C. Cinesi, L. Aeschbach, B. Yang, V. Dion, Contracting CAG/CTG repeats using the CRISPR-Cas9 nickase. *Nat. Commun.* **7**, 13272 (2016).

34. P. J. P. de Vree, E. de Wit, M. Yilmaz, M. van de Heijning, P. Klous, M. J. A. M. Verstegen, Y. Wan, H. Teunissen, P. H. L. Krijger, G. Geeven, P. P. Eijk, D. Sie, B. Ylstra, L. O. M. Hulsman, M. F. van Dooren, L. J. C. M. van Zutven, A. van den Ouweland, S. Verbeek, K. W. Van Dijk, M. Cornelissen, A. T. Das, B. Berkhout, B. Sikkema-Raddatz, E. van den Berg, P. van der Vlies, D. Weening, J. T. den Dunnen, M. Matusiak, M. Lamkanfi, M. J. L. Ligtenberg, P. ter Brugge, J. Jonkers, J. A. Foekens, J. W. Martens, R. van der Luijt, H. K. P. van Amstel, M. van Min, E. Splinter, W. de Laat, Targeted sequencing by proximity ligation for comprehensive variant detection and local haplotyping. *Nat. Biotechnol.* **32**, 1019–1025 (2014).

35. A. Rada-Iglesias, F. G. Grosveld, A. Papantonis, Forces driving the three-dimensional folding of eukaryotic genomes. *Mol. Syst. Biol.* **14**, e8214 (2018).

36. M. Merkenschlager, E. P. Nora, CTCF and cohesin in genome folding and transcriptional gene regulation. *Annu. Rev. Genomics Hum. Genet.* **17**, 17–43 (2016).

37. R. Adihe Lokanga, X.-N. Zhao, A. Entezam, K. Usdin, X inactivation plays a major role in the gender bias in somatic expansion in a mouse model of the fragile X-related disorders: Implications for the mechanism of repeat expansion. *Hum. Mol. Genet.* **23**, 4985–4994 (2014).

38. D. Wöhrle, U. Salat, H. Hameister, W. Vogel, P. Steinbach, Demethylation, reactivation, and destabilization of human fragile X full-mutation alleles in mouse embryocarcinoma cells. *Am. J. Hum. Genet.* **69**, 504–515 (2001).

39. G. De Michele, F. Cavalcanti, C. Criscuolo, L. Pianese, A. Monticelli, A. Filla, S. Cocozza, Parental gender, age at birth and expansion length influence GAA repeat intergenerational instability in the X25 gene: Pedigree studies and analysis of sperm from patients with Friedreich's ataxia. *Hum. Mol. Genet.* **7**, 1901–1906 (1998).

40. Y. Ghavi-Helm, A. Jankowski, S. Meiers, R. R. Viales, J. O. Korbel, E. E. M. Furlong, Highly rearranged chromosomes reveal uncoupling between genome topology and gene expression. *Nat. Genet.* **51**, 1272–1282 (2019).

41. A. R. Barutcu, P. G. Maass, J. P. Lewandowski, C. L. Weiner, J. L. Rinn, A TAD boundary is preserved upon deletion of the CTCF-rich Firre locus. *Nat. Commun.* **9**, 1444 (2018).

42. E. Rodríguez-Carballo, L. Lopez-Delisle, Y. Zhan, P. J. Fabre, L. Beccari, I. El-Idrissi, T. H. Nguyen Huynh, H. Ozadam, J. Dekker, D. Duboule, The *HoxD* cluster is a dynamic and resilient TAD boundary controlling the segregation of antagonistic regulatory landscapes. *Genes Dev.* **31**, 2264–2281 (2017).

43. V. Dion, Y. Lin, L. Hubert Jr, R. A. Waterland, J. H. Wilson, Dnmt1 deficiency promotes CAG repeat expansion in the mouse germline. *Hum. Mol. Genet.* **17**, 1306–1317 (2008).

44. D. Gaidatzis, A. Lerch, F. Hahne, M. B. Stadler, QuasR: Quantification and annotation of short reads in R. *Bioinformatics* **31**, 1130–1132 (2015).

45. B. Langmead, S. L. Salzberg, Fast gapped-read alignment with Bowtie 2. *Nat. Methods* **9**, 357–359 (2012).

46. P. V. Kharchenko, M. Y. Tolstorukov, P. J. Park, Design and analysis of ChIP-seq experiments for DNA-binding proteins. *Nat. Biotechnol.* **26**, 1351–1359 (2008).

47. Q. Li, J. B. Brown, H. Huang, P. J. Bickel, Measuring reproducibility of high-throughput experiments. *Ann. Appl. Stat.* **5**, 1752–1779 (2011).

48. C. S. Ross-Innes, R. Stark, A. E. Teschendorff, K. A. Holmes, H. R. Ali, M. J. Dunning, G. D. Brown, O. Gojis, I. O. Ellis, A. R. Green, S. Ali, S.-F. Chin, C. Palmieri, C. Caldas, J. S. Carroll, Differential oestrogen receptor binding is associated with clinical outcome in breast cancer. *Nature* **481**, 389–393 (2012).

49. M. I. Love, W. Huber, S. Anders, Moderated estimation of fold change and dispersion for RNA-seq data with DESeq2. *Genome Biol.* **15**, 550 (2014).

50. F. P. A. David, J. Delafontaine, S. Carat, F. J. Ross, G. Lefebvre, Y. Jarosz, L. Sinclair, D. Noordermeer, J. Rougemont, M. Leleu, HTSstation: A web application and open-access libraries for high-throughput sequencing data analysis. *PLOS ONE* **9**, e85879 (2014).

51. D. Noordermeer, M. Leleu, E. Splinter, J. Rougemont, W. De Laat, D. Duboule, The dynamic architecture of Hox gene clusters. *Science* **334**, 222–225 (2011).

52. C. Walter, D. Schuetzmann, F. Rosenbauer, M. Dugas, Benchmarking of 4C-seq pipelines based on real and simulated data. *Bioinformatics* **35**, 4938–4945 (2019).

53. A. McKenna, M. Hanna, E. Banks, A. Sivachenko, K. Cibulskis, A. Kernytsky, K. Garimella, D. Altshuler, S. Gabriel, M. Daly, M. A. DePristo, The genome analysis toolkit: A MapReduce framework for analyzing next-generation DNA sequencing data. *Genome Res.* **20**, 1297–1303 (2010).

54. H. Li, A statistical framework for SNP calling, mutation discovery, association mapping and population genetical parameter estimation from sequencing data. *Bioinformatics* **27**, 2987–2993 (2011).

55. M. Martin, Cutadapt removes adapter sequences from high-throughput sequencing reads. *EMBnet.J.* **17**, 10–12 (2011).

56. A. Dobin, C. A. Davis, F. Schlesinger, J. Drenkow, C. Zaleski, S. Jha, P. Batut, M. Chaisson, T. R. Gingeras, STAR: Ultrafast universal RNA-seq aligner. *Bioinformatics* **29**, 15–21 (2013).

57. D. W. Huang, B. T. Sherman, R. A. Lempicki, Bioinformatics enrichment tools: Paths toward the comprehensive functional analysis of large gene lists. *Nucleic Acids Res.* **37**, 1–13 (2009).

58. D. W. Huang, B. T. Sherman, R. A. Lempicki, Systematic and integrative analysis of large gene lists using DAVID bioinformatics resources. *Nat. Protoc.* **4**, 44–57 (2009).

59. H. Li, R. Durbin, Fast and accurate long-read alignment with Burrows-Wheeler transform. *Bioinformatics* **26**, 589–595 (2010).

60. H. Yao, K. Brick, Y. Evrard, T. Xiao, R. D. Camerini-Otero, G. Felsenfeld, Mediation of CTCF transcriptional insulation by DEAD-box RNA-binding protein p68 and steroid receptor RNA activator SRA. *Genes Dev*. **24**, 2543–2555 (2010).

61. H. J. G. van de Werken, G. Landan, S. J. B. Holwerda, M. Hoichman, P. Klous, R. Chachik, E. Splinter, C. Valdes-Quezada, Y. Oz, B. A. M. Bouwman, M. J. A. M. Verstegen, E. de Wit, A. Tanay, W. de Laat, Robust 4C-seq data analysis to screen for regulatory DNA interactions. *Nat. Methods* **9**, 969–972 (2012).
